# Identification of Conserved MEL-28/ELYS Domains with Essential Roles in Nuclear Assembly and Chromosome Segregation

**DOI:** 10.1371/journal.pgen.1006131

**Published:** 2016-06-24

**Authors:** Georgina Gómez-Saldivar, Anita Fernandez, Yasuhiro Hirano, Michael Mauro, Allison Lai, Cristina Ayuso, Tokuko Haraguchi, Yasushi Hiraoka, Fabio Piano, Peter Askjaer

**Affiliations:** 1 Andalusian Center for Developmental Biology (CABD), CSIC/Junta de Andalucia/Universidad Pablo de Olavide, Seville, Spain; 2 Biology Department, Fairfield University, Fairfield, Connecticut, United States of America; 3 Graduate School of Frontier Biosciences, Osaka University, Suita, Japan; 4 Advanced ICT Research Institute Kobe, National Institute of Information and Communications Technology, Kobe, Japan; 5 Department of Biology and Center for Genomics and Systems Biology, New York University, New York, New York, United States of America; 6 New York University, Abu Dhabi, United Arab Emirates; National Institute of Diabetes and Digestive and Kidney Diseases, UNITED STATES

## Abstract

Nucleoporins are the constituents of nuclear pore complexes (NPCs) and are essential regulators of nucleocytoplasmic transport, gene expression and genome stability. The nucleoporin MEL-28/ELYS plays a critical role in post-mitotic NPC reassembly through recruitment of the NUP107-160 subcomplex, and is required for correct segregation of mitotic chromosomes. Here we present a systematic functional and structural analysis of MEL-28 in *C*. *elegans* early development and human ELYS in cultured cells. We have identified functional domains responsible for nuclear envelope and kinetochore localization, chromatin binding, mitotic spindle matrix association and chromosome segregation. Surprisingly, we found that perturbations to MEL-28’s conserved AT-hook domain do not affect MEL-28 localization although they disrupt MEL-28 function and delay cell cycle progression in a DNA damage checkpoint-dependent manner. Our analyses also uncover a novel meiotic role of MEL-28. Together, these results show that MEL-28 has conserved structural domains that are essential for its fundamental roles in NPC assembly and chromosome segregation.

## Author Summary

Most animal cells have a nucleus that contains the genetic material: the chromosomes. The nucleus is enclosed by the nuclear envelope, which provides a physical barrier between the chromosomes and the surrounding cytoplasm, and enables precisely controlled transport of proteins into and out of the nucleus. Transport occurs through nuclear pore complexes, which consist of multiple copies of ~30 different proteins called nucleoporins. Although the composition of nuclear pore complexes is known, the mechanisms of their assembly and function are still unclear. We have analyzed the nucleoporin MEL-28/ELYS through a systematic dissection of functional domains both in the nematode *Caenorhabditis elegans* and in human cells. Interestingly, MEL-28/ELYS localizes not only to nuclear pore complexes, but is also associated with chromosomal structures known as kinetochores during cell division. Our studies have revealed that even small perturbations in MEL-28/ELYS can have dramatic consequences on nuclear pore complex assembly as well as on separation of chromosomes during cell division. Surprisingly, inhibition of MEL-28/ELYS causes cell-cycle delay, suggesting activation of a cellular surveillance system for chromosomal damages. Finally, we conclude that the structural domains of MEL-28/ELYS are conserved from nematodes to humans.

## Introduction

Metazoans have an open mitosis, in which the nuclear envelope (NE) disassembles during prophase to allow chromosome segregation and then reassembles around condensing chromosomes at anaphase [1]. During this process, the nuclear pore complexes (NPCs) are disassembled then rapidly reconstructed. ELYS, a large AT-hook domain protein, is essential for the late-mitosis rebuilding of the NPC [2]. ELYS is the first NPC component to associate with chromatin at the end of mitosis [3, 4] and this association is required for the recruitment of the NUP107-160 subcomplex of the NPC, which in turn recruits vesicles containing the membrane-bound nucleoporins POM121 and NDC1 [4]. Thus ELYS binding to chromatin represents the first step in the post-mitotic building of the pore, and all other steps in its manufacture are dependent on this ELYS/chromatin interaction.

ELYS was originally identified in a cDNA subtraction screen seeking genes expressed at high levels in the mouse embryonic sac [5]. Mouse *elys* knockouts die in the preimplantation stage because of cell death within the inner cell mass [6]. ELYS function is essential in all metazoa and is particularly important in rapidly dividing cells [7, 8]. In *C*. *elegans*, the orthologous MEL-28 protein dynamically localizes to the nucleoplasm and NPC at interphase and then at the kinetochore and spindle at metaphase [9, 10]. Consistent with its localization pattern, embryos that lack *mel-28* function have severe defects with NE function, mitotic spindle assembly and chromosome segregation and are unviable.

The ELYS/chromatin interaction has been studied extensively *in vitro* using *Xenopus* cell extracts. ELYS binds to chromatin during interphase but not at metaphase [11], when it instead associates with the spindle and kinetochore [12]. Chromatin immobilization assays have shown that the most C-terminal fragment of ELYS, corresponding to amino acids (aa.) 2281–2408, is sufficient for chromatin binding. This region includes the AT hook, a motif that binds to AT-rich DNA. However the aa. 2281–2408 fragment with a mutated AT hook and a C-terminal fragment that excludes the AT hook (aa. 2359–2408) also bound to chromatin [4]. A nucleosome binding assay showed that a large C-terminal fragment that includes the AT hook (aa. 2281–2408) was sufficient to bind to nucleosomes, whereas a piece that includes just the AT hook (aa. 2281–2358) or just the region C-terminal to the AT hook (aa. 2359–2408) could not bind to nucleosomes [13]. Additionally, incubation of *Xenopus* extracts with the C-terminal 208-aa. fragment of ELYS prevented native ELYS from binding to sperm chromatin and also prevented the recruitment of other nucleoporins to the nuclear rim, phenocopying the *elys* loss-of-function phenotype [11]. However, introducing a C-terminal fragment with a mutated AT hook does not disrupt nuclear pore assembly and is less effective at outcompeting the endogenous ELYS from binding to chromatin [4]. These *in vitro* experiments suggest that both the AT hook and other domains of the C terminus are important for the ELYS/chromatin interaction and the subsequent rebuilding of the NPC.

The ELYS/chromatin association has also been studied using mouse *in vitro* fertilization. During fertilization in mice, sperm chromatin is rebuilt *de novo* using histones present in the oocyte. Experiments using *in vitro* fertilized mouse oocytes depleted of histones showed that ELYS does not localize to the NE of the sperm pronucleus in the absence of histones, which in turn prevents the recruitment of other nucleoporins [14]. ELYS can be artificially targeted to the NE in the absence of histones by fusing it with a domain from an inner NE protein. This chimeric ELYS protein not only localizes to the NE but also recruits the other nucleoporins. This suggests that ELYS binding to chromatin is required for its localization to the nuclear rim, which in turn allows the remainder of the nuclear pore to be built.

The overall architecture of MEL-28/ELYS is similar throughout the metazoa (see schematic representations in Figs 2C and 7B). All metazoan MEL-28/ELYS homologs include an N-terminal β-propeller domain, a central α-helical domain, and a C-terminal domain that includes at least one AT hook. Crystal structure determination of the N-terminal domain of mammalian ELYS showed that it forms a seven bladed β-propeller structure with an extra loop decorating each of the propeller blades [15]. In human cells, the N-terminal 1018 amino acids of ELYS (which includes the β-propeller domain and the central α-helical domain but not the C-terminal AT hook) is sufficient to localize the protein to NPCs [15]. Mutational disruption of the conserved loop on blade 6 of the β-propeller domain (“loop2”) prevents the 1–1018 aa. fragment from localizing to the nuclear rim.

Despite the interest in defining the functional domains of MEL-28/ELYS, until now there have been no studies in which the phenotypic consequences of disrupting specific domains have been studied in developing animals. In this work, we have dissected the MEL-28 protein and studied its localization and function in live *C*. *elegans* embryos. We have identified regions of MEL-28 required for its roles in meiosis as well as in chromatin binding and post-mitotic nuclear pore construction. Our parallel studies in HeLa cells show that the domains required for proper localization in *C*. *elegans* are conserved in human ELYS, suggesting that conclusions from functional analyses of MEL-28 in *C*. *elegans* are broadly applicable to vertebrate ELYS.

## Results

### MEL-28 is required for meiotic chromosome segregation

We previously reported that *C*. *elegans* MEL-28 is broadly expressed [10]. However, a promoter study of 127 genes in *C*. *elegans* embryos suggested that MEL-28 is highly enriched in the intestinal E lineage ~200 min after fertilization [16]. We therefore revisited MEL-28 expression to analyze it in greater detail. Immunofluorescence analysis detected similar levels of MEL-28 in nuclei of all embryonic cells (S1A Fig) and all postembryonic tissues (S1B Fig). Next, using CRISPR-Cas9 technology [17], we generated a GFP knock-in *mel-28* allele to analyze the expression of endogenous MEL-28 by live microscopy. Similar to the observations with antibodies against MEL-28, GFP::MEL-28 localized to the NE in all cell types during embryonic and larval development and in adults (S1C Fig). Thus, we conclude that MEL-28 is ubiquitously expressed throughout *C*. *elegans* development.

**Fig 1:**
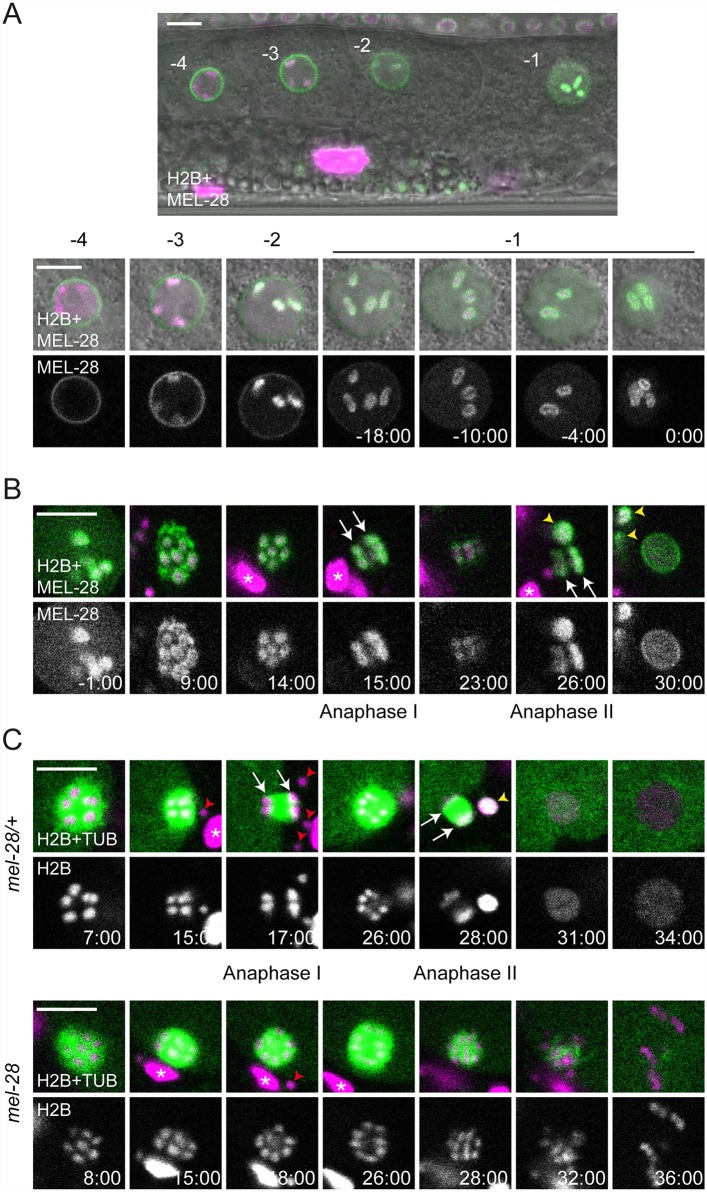
MEL-28 is essential for female meiosis. (A) GFP::MEL-28 (green in merged images) was expressed in oocytes and accumulated at kinetochores of meiotic chromosomes (visualized with mCherry::HisH2B; magenta in merge). Shown are the four most proximal oocytes where position -1 is immediately next to the spermatheca. The -1 oocyte was observed every two minutes until germinal vesicle breakdown. (B) GFP::MEL-28 associated with chromosomes throughout meiosis I and II and accumulated at the NE at pronuclear formation. (C) Chromosomes (magenta) and meiotic spindles (green) were observed *in utero*. Anaphase I and II were characterized by abundant microtubules between segregating chromosomes in control *mel-28/+* animals (top) whereas chromosomes failed to segregate in homozygous *mel-28* mutants (bottom). White arrows point to segregating chromosomes. Red arrowheads and white asterisks mark sperm and somatic nuclei, respectively, outside the fertilized oocyte; yellow arrowheads indicate polar bodies. Time is indicated relative to germinal vesicle breakdown (min:sec). Scale bars, 5 μm.

MEL-28 strongly accumulated on condensed oocyte chromosomes (S1C Fig; [9, 18]). Moreover, we noted during our initial studies of *mel-28* mutant or RNAi-treated embryos that formation and migration of the maternal pronucleus was often more severely affected than the paternal pronucleus [9, 10]. Based on these observations we speculated that MEL-28 might have important functions in meiosis. *C*. *elegans* oocytes are arranged in a linear fashion in the proximal part of the gonad, where each oocyte is numbered relative to the spermatheca (-1, -2, -3, etc.) [19]. The -1 oocyte completes maturation including germinal vesicle breakdown immediately before ovulation and fertilization triggers rapid progression through meiosis I and II. To examine these processes we performed live *in utero* recordings of animals expressing GFP::MEL-28 and mCherry::HisH2B. In the -4 oocyte, MEL-28 localized to the NE and was absent from condensed chromosomes (Fig 1A). In the -3 and -2 oocytes MEL-28 gradually moved away from the NE and accumulated uniformly on meiotic chromosomes. Later, in the -1 oocyte MEL-28 redistributed to cover the surface of meiotic chromosomes (Fig 1A; S1 Video), in some cases completely enclosing the chromosomes and in other cases similar to the “cup-shaped” localization of kinetochore proteins, such as KNL-1 and KNL-3 [20]. The association of MEL-28 with chromosomes persisted throughout meiosis I and II until pronuclear formation ~30 minutes after germinal vesicle breakdown (Fig 1B; S1 Video). The localization pattern of MEL-28 suggested a possible role during segregation of meiotic chromosomes, similar to the situation in mitosis [9, 10]. We therefore analyzed *mel-28(t1684)* embryos expressing GFP::β-tubulin and mCherry::HisH2B. *mel-28(t1684)* encodes a premature termination codon at aa. 766 and behaves like a strong loss-of-function of MEL-28, presumably due to nonsense-mediated mRNA decay [10]. Maternal contribution enables homozygous *mel-28(t1684)* hermaphrodites to develop until adulthood but they produce only unviable embryos (hereafter referred to as *mel-28* embryos, whereas embryos produced by heterozygous siblings are referred to as control or *mel-28/+* embryos) with severe NE assembly defects [10]. Strikingly, in *mel-28* embryos chromosomes failed to segregate in anaphase I (n = 5/6 embryos) and anaphase II (n = 4/6) and, consequently, *mel-28* embryos had either no (n = 4/6) or a single (n = 2/6) polar body, whereas control embryos had two polar bodies (n = 6/6; Fig 1C; S2 Video). In addition, chromosomes in *mel-28* embryos were not organized in a pronucleus but appeared scattered in the cytoplasm (Fig 1C; 36:00). To our knowledge, this is the first report describing the involvement of MEL-28/ELYS in meiosis, expanding previously described MEL-28 functions and establishing an important role in chromosome segregation during both meiosis and mitosis.

### The MEL-28 N-terminus is required for NPC association

**Fig 2:**
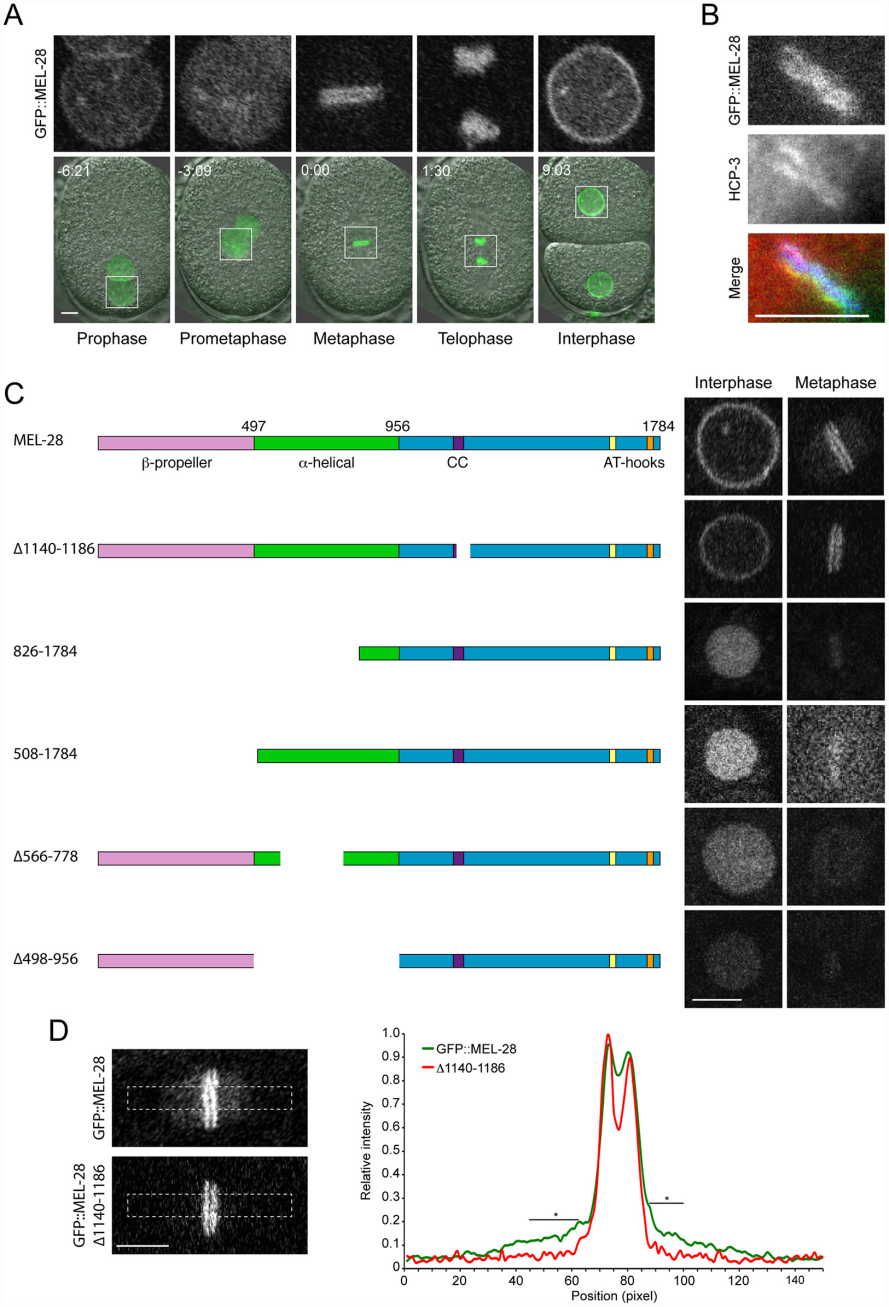
MEL-28 N-terminal domains are required for NPC and kinetochore localization. (A) Still images from time-lapse recording of embryo carrying a GFP insertion into the endogenous *mel-28* locus. Time is indicated relative to anaphase onset (min:sec). (B) Metaphase plate of early embryo expressing GFP::MEL-28 (green in merge) analyzed by immunofluorescence with a specific antibody against HCP-3/CENP-A (red in merge) and Hoechst (blue in merge) to visualize chromosomes. MEL-28 localized to kinetochores, which appear as lines on both sides of the chromosomes. (C) Cropped images from embryos expressing different MEL-28 truncations fused to GFP. Except GFP::MEL-28 and GFP::MEL-28^Δ1140–1186^ embryos, all embryos also expressed un-tagged endogenous MEL-28. Purple boxes in MEL-28 cartoons indicate a putative coiled-coil domain (aa. 1127–1160) whereas yellow (aa. 1630–1642) and orange (aa. 1746–1758) boxes indicate AT-hook sequences: their homology to the consensus AT-hook sequence is low and high, respectively. (D) Cropped images from metaphase embryos expressing GFP::MEL-28 or GFP::MEL-28^Δ1140–1186^. Images were processed identically to facilitate visualization of full-length GFP::MEL-28 associated with the mitotic spindle. Signal intensities in boxed areas were quantified in raw images, normalized and plotted (n = 5, GFP::MEL-28; n = 2, GFP::MEL-28^Δ1140–1186^). * p<0.05 by unpaired two-tailed t-test. Scale bars, 5 μm.

To characterize which regions of MEL-28 are required for its different functions, we examined full-length and truncated versions of MEL-28 fused to GFP and tracked their localization in live *C*. *elegans* embryos. While most transgenes are expressed (S2 Fig; S4 Fig), some exhibit localization patterns distinct from full-length MEL-28 (see below). During interphase full-length MEL-28 was mainly localized to the NE but was also found in the nucleoplasm (Fig 2A; S3 Video; S9 Video). In prophase and prometaphase, MEL-28 left the NE before complete NE breakdown and associated to the condensing chromosomes. By metaphase, MEL-28 appeared as two lines parallel to the metaphase plate, resembling the characteristic pattern of holocentric kinetochore proteins, and less abundantly to the area of the mitotic spindle (Fig 2A–2D). During anaphase, MEL-28 associated to decondensing chromosomes, and re-localized to reforming NE in telophase (Fig 2A; S3 Video).

We next analyzed a putative coiled-coil domain placed in the central part of the protein and which might be engaged in protein—protein interactions. However, GFP::MEL-28 lacking aa. 1140–1186 localized similarly to full-length MEL-28 (Fig 2C; S3 Video). During interphase MEL-28^Δ1140–1186^ was enriched at the NE and shuttled to kinetochores in mitosis whereas reduced signal was observed at the mitotic spindle (Fig 2D). Moreover, expression of GFP::MEL-28^Δ1140–1186^ completely rescued the embryonic lethality of *mel-28* mutant embryos (Table 1). This demonstrated that the putative coiled-coil domain as well as enrichment at the mitotic spindle is dispensable for MEL-28 function.

**Table 1:** Rescue efficiencies by MEL-28 fragments.

MEL-28 fragment^a^	Promoter	*n*^b^	Embryonic lethality^d^	S.D.^g^
-	-	964	100%	0
GFP::MEL-28	Constitutive	372	7.1%^e^	10.8
GFP::MEL-28^Δ1140–1186^	Constitutive	632	1%	1.5
GFP::MEL-28^1-1744^	Constitutive	1424	65.9%	21.4
GFP::MEL-28^1-1629^	Constitutive	1384	99.4%	1.3
GFP::MEL-28^508-1784^	Constitutive	240	100%	0
GFP::MEL-28^Δ498–956^	Constitutive	202	100%	0
MEL-28^loop2mut^::GFP	Constitutive	512	100%	0
GFP::MEL-28	Inducible	328^c^	No^f^	-
GFP::MEL-28^1-1601^	Inducible	255^c^	Yes^f^	-
GFP::MEL-28^Δ1239–1728^	Inducible	117^c^	Yes^f^	-

^a^ GFP::MEL-28 fusions were expressed in *mel-28(t1684)* mutants.

^b,c^ The number of embryos^b^ or worms^c^ analyzed is indicated.

^d^ Percentage of unhatched embryos.

^e^ This strain was also homozygous for *unc-32(e189)*, which may have influenced the incomplete rescue.

^f^ Qualitative analysis was done for fragments under control of an inducible promoter due to heterogeneity of the expression.

^g^ Standard deviation.

Recently, Bilokapic and Schwartz found that the N-terminal half of ELYS containing the β-propeller and α-helical domains localized to the NE in HeLa cells [15]. However, the relevance of these domains has not been analyzed in the context of full-length MEL-28/ELYS. We first deleted the β-propeller and most of the α-helical domain (GFP::MEL-28^826-1784^) and found that both NE localization during interphase and kinetochore localization in mitosis were abrogated (Fig 2C). Instead, the truncated protein was found in the nucleoplasm and weakly associated with chromosomes during interphase and metaphase, respectively (note that kinetochore localization appears as two parallel lines whereas a single line reflects more uniform chromosome association). Similar mis-localization was observed on deletion of aa. 1–507 (GFP::MEL-28^508-1784^) or aa. 498–956 (GFP::MEL-28^Δ498–956^), whereas deletion of aa. 566–778 (GFP::MEL-28^Δ566–778^) also abolished the weak association to mitotic chromosomes (Fig 2C). Together, these results demonstrate that both the β-propeller and the α-helical domain are required for targeting MEL-28 to NPCs and to kinetochores. All four N-terminally truncated MEL-28 proteins accumulated in the nucleus in interphase, suggesting that the C-terminal unstructured domain of MEL-28 contains one or more nuclear localization signals (NLS’s; see below).

Finally, we assessed whether the truncations in the β-propeller and α-helical domains interfered with MEL-28 function. As expected from the severe mis-localization, ectopic expression of any of the four MEL-28 truncations failed to restore viability of *mel-28* embryos (Table 1), suggesting that the localization of MEL-28 to NPCs and kinetochores is essential to MEL-28 function. We conclude from these experiments that the N terminus of MEL-28 is required for proper MEL-28 localization and functions. Whereas its importance for NPC localization is concordant with data on ELYS our experiments revealed a novel role in kinetochore association.

### MEL-28 loop2 is required during meiosis and mitosis

**Fig 3:**
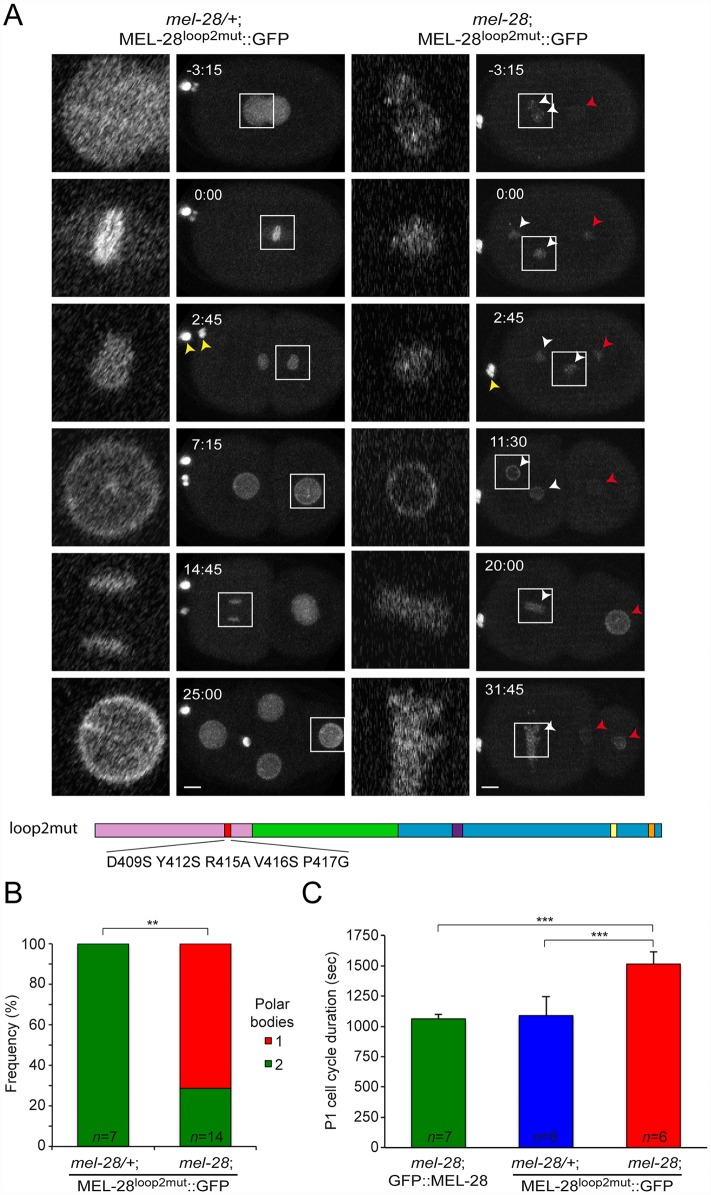
MEL-28 loop2 is required during meiosis and mitosis. (A) Still images from time-lapse recordings of control (left) and *mel-28* (right) embryos expressing MEL-28^loop2mut^::GFP. Note the presence of two polar bodies in the left embryo but only a single polar body in the right embryo (yellow arrowheads). Concordantly, two oocyte-derived pronuclei were observed in the right embryo (white arrowheads). Red arrowheads indicate sperm-derived chromosomes. Whole-embryo images are max projections; inserts are single confocal sections. Scale bars, 5 μm. (B) Frequency of embryos with a single or two polar bodies. ** p<0.01 by Fisher exact test. (C) Timing from P0 division to P1 division is significantly delayed in *mel-28* embryos expressing MEL-28^loop2mut^::GFP. *** p<0.001 by unpaired two-tailed t-test.

Bilokapic and Schwartz identified through protein crystallization and sequence alignments two conserved loops (loop1 and loop2) on the surface of the β-propeller of ELYS [15]. When they substituted 5 aa. within loop2 the structural fold of the β-propeller was maintained but NPC localization of the N-terminal half of ELYS (aa. 1–1018) fused to GFP was abrogated in HeLa cells. To test the relevance of loop2 in the context of full-length protein we introduced the equivalent aa. substitutions in MEL-28 (D409S/Y412S/R415A/V416S/P417G; MEL-28^loop2mut^; Fig 3A). In *mel-28/+* embryos MEL-28^loop2mut^::GFP localized normally during interphase and mitosis (Fig 3A, left panels; compare with wild type GFP::MEL-28 in Fig 2A; S4 Video; S3 Fig), suggesting that loop2 residues are not essential for association of full-length MEL-28 with NPCs or kinetochores. However, MEL-28^loop2mut^::GFP was not able to substitute for endogenous MEL-28: *mel-28* embryos expressing MEL-28^loop2mut^::GFP were unviable (Table 1) and had frequent meiosis defects as evidenced by failure in polar body extrusion and presence of multiple female pronuclei (Fig 3A, right panels; S4 Video; Fig 3B). Moreover, pronuclei were abnormally small, contained less MEL-28^loop2mut^::GFP and did not position properly. In 83% of *mel-28*; MEL-28^loop2mut^::GFP embryos (n = 10/12) female and male pronuclei did not meet before the first mitotic division. Instead, only the male pronucleus was positioned between the centrosomes, whereas female pronuclei exhibited shorter migration and remained in the anterior of the embryo. During mitosis chromosomes failed to congress to the metaphase plate (Fig 3A; 0:00) and severe segregation defects were observed (Fig 3A; 20:00–31:45). We also noticed alterations in cell cycle timing, in particular for the posterior P1 blastomere at the two-cell stage. In *mel-28*; GFP::MEL-28 and *mel-28/+*; MEL-28^loop2mut^::GFP embryos the cell cycle of P1 lasted ~1075 sec, whereas it lasted ~1513 sec (41% delay) in *mel-28* embryos expressing MEL-28^loop2mut^::GFP (Fig 3C). Other frequent defects included cleavage furrow regression (37%; n = 6/16) and abnormal positioning of cells within the eggshell (53%; n = 8/15).

**Fig 4:**
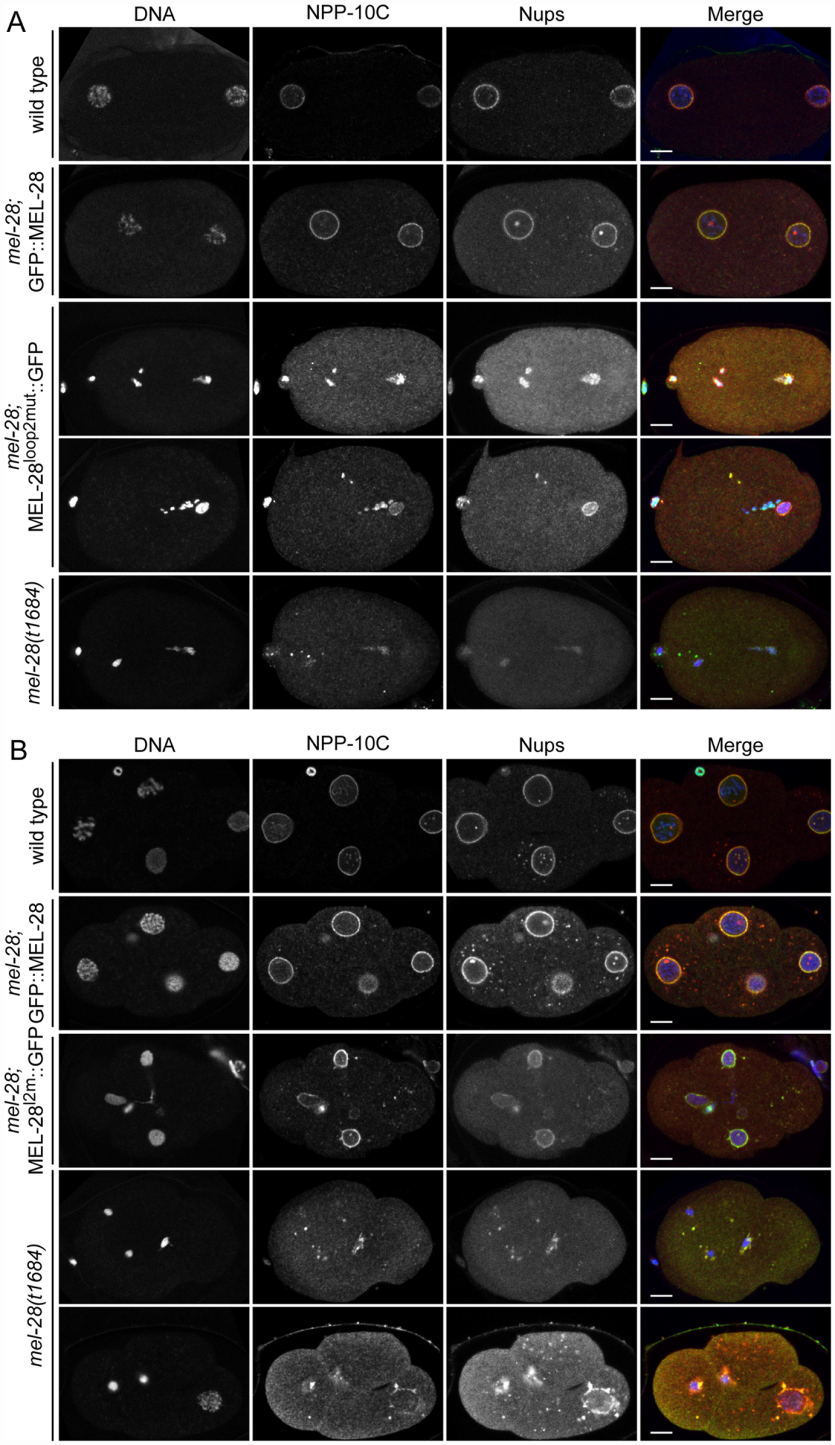
Mutation of MEL-28 loop2 impairs chromosome segregation. One-cell stage (A) and 4-cell stage (B) embryos from *mel-28* mutants expressing either GFP::MEL-28 or MEL-28^loop2mut^::GFP were compared with wild type and *mel-28* embryos by immunofluorescence. Embryos were analyzed with Hoechst (blue in merge), a specific antibody against NPP-10C/NUP96 (green in merge) and mAb414 recognizing multiple nups (red in merge). Scale bars, 5 μm.

To analyze if the conserved loop2 is required for MEL-28’s role in NPC assembly we performed immunofluorescence on *mel-28*; MEL-28^loop2mut^::GFP embryos and compared them with wild type, *mel-28*, and *mel-28*; GFP::MEL-28 embryos. One-cell and four-cell stage embryos were analyzed for meiotic and mitotic defects, respectively, using mAb414 to visualize multiple Nups and specific antibodies against NPP-10C/NUP96, which is a component of the NUP107 complex [21]. Uniform peripheral signal was observed at pronuclei of wild type and *mel-28*; GFP::MEL-28 one-cell stage embryos, whereas fragmented pronuclei with inconsistent Nup signal was detected in *mel-28*; MEL-28^loop2mut^::GFP and *mel-28* embryos (Fig 4A). Analysis of four-cell stage *mel-28*; MEL-28^loop2mut^::GFP embryos confirmed the defects in chromosome segregation observed by live imaging and revealed that although nuclei with peripheral Nup localization are formed, these are smaller than in wild type and *mel-28*; GFP::MEL-28 embryos (Fig 4B). The NE phenotypes in *mel-28*; MEL-28^loop2mut^::GFP embryos were less severe when compared to *mel-28* embryos. As previously reported, nuclear reformation and NPC assembly was strongly inhibited in *mel-28* embryos although a few cells had larger nuclei with irregular NE-structure (Fig 4B; bottom *mel-28* embryo).

From these data we conclude that MEL-28’s loop2 is essential for correct chromosome segregation both in meiosis and mitosis but not strictly required for post mitotic NPC assembly, nor for incorporation into the NE.

### Identification of MEL-28 nuclear localization and chromatin association domains

**Fig 5:**
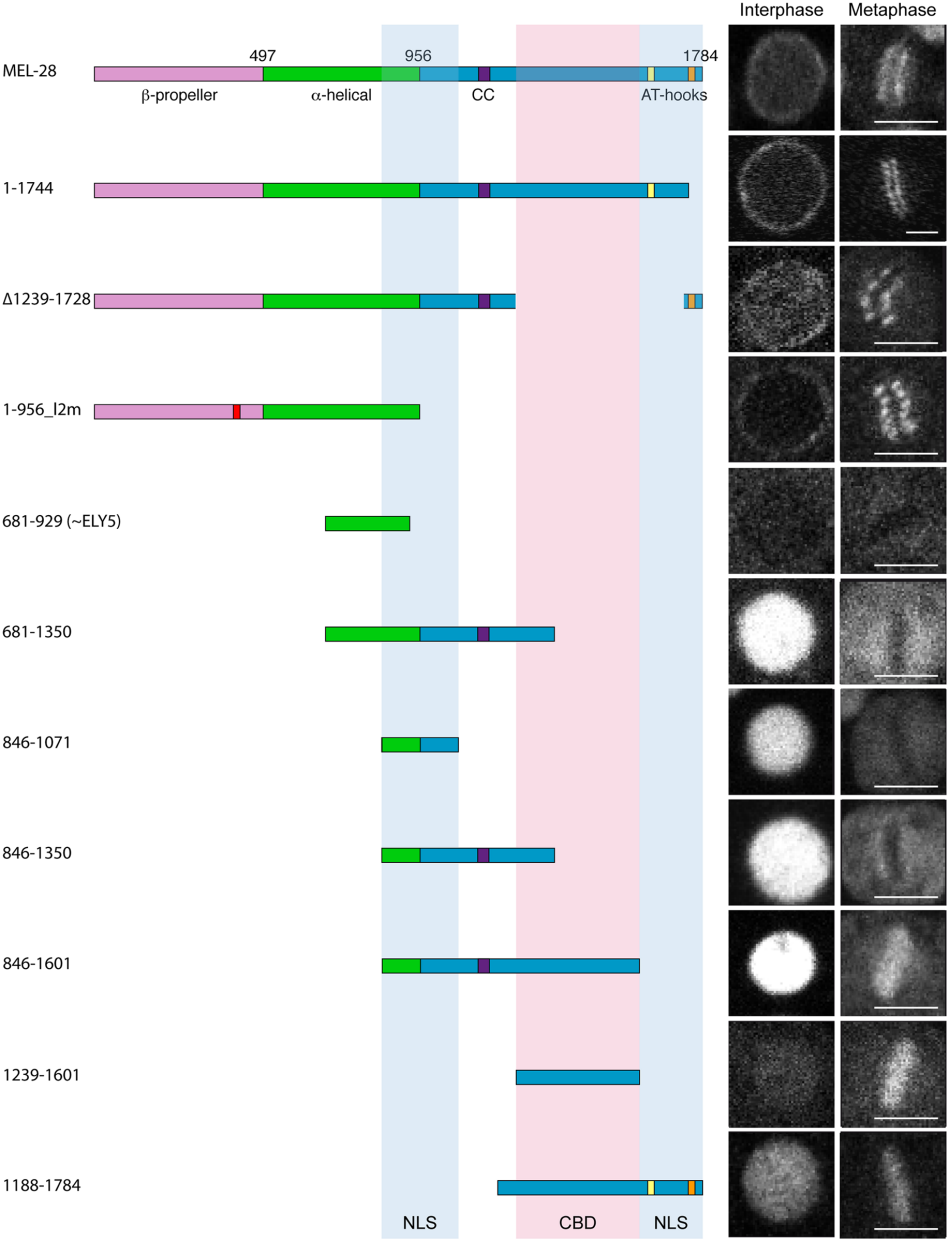
Identification of MEL-28 chromatin binding domain and nuclear localization signals. Cropped images from embryos expressing different MEL-28 truncations fused to GFP. Except GFP::MEL-28^1-1744^ and GFP::MEL-28^1188-1784^, fusion proteins were expressed from the *hsp-16*.*41* promoter in gastrulating embryos. Excluding GFP::MEL-28^1-1744^ embryos, all embryos also expressed un-tagged endogenous MEL-28. Truncations containing MEL-28 residues 846–1071 and/or residues 1602–1784 (blue shading) were efficiently imported whereas truncations containing residues 1239–1601 (red shading) associated with chromatin in mitosis. Scale bars, 3 μm.

The observation that perturbations in MEL-28’s N-terminal half do not prevent nuclear accumulation of MEL-28 prompted us to analyze the C-terminus for functional domains. We first expressed GFP::MEL-28^1-1744^, which lacks 40 aa. from the C-terminal end including one of the two AT-hook motifs. This short truncation did not interfere with MEL-28 localization in interphase nor during mitosis (Fig 5; S5 Video). However, expression of GFP::MEL-28^1-1744^ rescued lethality in only ~35% of *mel-28* embryos (Table 1), indicating that the C-terminal AT hook of MEL-28 contributed significantly to MEL-28 activity. Next, we deleted aa. 1239–1728, including the other AT-hook motif. This reduced slightly the NE accumulation at interphase (Fig 5; GFP::MEL-28^Δ1239–1728^; S6 Video). Importantly, expression of GFP::MEL-28^Δ1239–1728^ was not able to rescue the embryonic lethality of *mel-28* embryos (Table 1), which suggests that there are domains within this region required for MEL-28 function. Despite several attempts, we were unable to express a MEL-28 aa. 1–956 fragment consisting of wild type β-propeller and α-helical domains (S4 Fig). In contrast, a similar fragment, but with the five aa. substitutions in loop2 described above was efficiently expressed (MEL-28^1-956_l2m^::GFP; S7 Video). MEL-28^1-956_l2m^::GFP localized to the cytoplasm and NE, but its relative NE accumulation compared to kinetochore localization was dramatically reduced (S3 Fig). As expected, expression of MEL-28^1-956_l2m^::GFP did not rescue the embryonic lethality of *mel-28* embryos (Table 1). Taken together with the results presented in Fig 2, we conclude that although the N-terminal β-propeller and α-helical domains are the main determinants for NPC and kinetochore localization, the C-terminal portion of MEL-28 also contributes significantly.

A divergent ~300 aa. MEL -28/ELYS homolog termed ELY5 was recently identified in several fungi [22, 23]. Although our experiments presented above would suggest that the part of MEL-28 equivalent to ELY5 (identified as aa. 696–927 by [24]) does not contain the domains required for NPC localization we nevertheless expressed a fragment containing aa. 681–929 fused to GFP. As expected, this fragment did not localize to the NE or to kinetochores but showed instead diffuse cytoplasmic signal throughout the cell cycle (Fig 5; GFP::MEL-28^681-929^; S2B Fig).

We next expressed a series of overlapping fragments from aa. 681 to the C-terminal end. All fragments that contained aa. 846–1071 accumulated efficiently in the nucleus (Fig 5; GFP::MEL-28^681-1350^, GFP::MEL-28^846-1071^, GFP::MEL-28^846-1350^, and GFP::MEL-28^846-1601^; S4A Fig; GFP::MEL-28^846-1167^; S8 Video). A shorter fragment consisting of aa. 846–956 behaved similarly to free GFP (S4A Fig; GFP::MEL-28^846-956^). Nuclear accumulation was also detected for GFP::MEL-28^1188-1784^, but not for GFP::MEL-28^1161-1601^ or GFP::MEL-28^1239-1601^ (Fig 5; S4A Fig). These observations are consistent with MEL-28 having at least two NLS’s mapping to the regions 846–1071 and 1601–1784. Moreover, using the NLS prediction software “cNLS Mapper” [25] we identified several putative mono- and bipartite NLSs in these regions: two in the central region (aa. 942–970 and 1033–1062 with scores 5.9 and 5.2, respectively) and three in the C-terminal region (aa. 1606–1636, 1682–1709 and 1741–1773 with scores 5.7, 7.4 and 5.3, respectively). Analysis of these C-terminal fragments also revealed that aa. 1239–1601 confer strong chromatin binding during mitosis (Fig 5).

### The AT-hook domain is dispensable for MEL-28 localization, but essential for its functions

**Fig 6:**
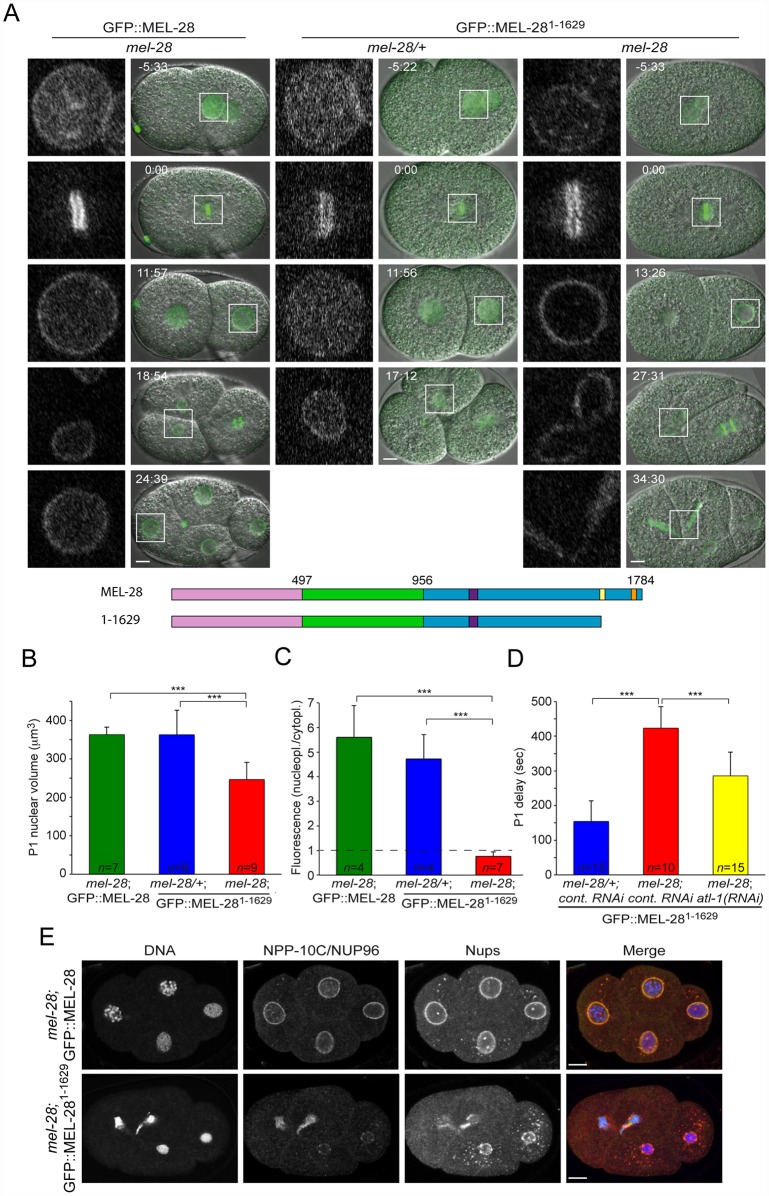
The AT-hook domain of MEL-28 is required for nuclear growth, chromosome segregation and cell cycle timing. (A) Still images from time-lapse recordings of control (middle) and *mel-28* (right) embryos expressing GFP::MEL-28^1-1629^ as well as a *mel-28* embryo expressing GFP::MEL-28 (left). Note defective chromosome segregation in the right embryo. Scale bars, 5 μm. Nuclear growth (B) and distribution of GFP fusion protein between nucleoplasm and cytoplasm (C) was specifically reduced in *mel-28* embryos expressing GFP::MEL-28^1-1629^. Measurements were performed on fully-grown P1 nuclei. (D) Asynchrony between division of AB and P1 blastomeres was significantly delayed in *mel-28* embryos expressing GFP::MEL-28^1-1629^; this delay was partially reduced by depletion of ATL-1. (E) Four-cell stage embryos from *mel-28* mutants expressing either GFP::MEL-28 or GFP::MEL-28^1-1629^ were analyzed with Hoechst (blue in merge), a specific antibody against NPP-10C/NUP96 (green in merge) and mAb414 recognizing multiple nups (red in merge). Scale bars, 5 μm. *** p<0.001 by unpaired two-tailed t-test.

Comparing the behavior of GFP::MEL-28^1239-1601^ and GFP::MEL-28^1188-1784^ indicated that MEL-28’s two AT hooks are not required for chromatin association, at least during mitosis (Fig 5). Moreover, *in vitro* binding experiments found no difference in chromatin affinity between recombinant peptides that contained either the C-terminal 128 aa of *Xenopus* ELYS including the single ELYS AT hook or a variant with mutated AT hook although the former was more efficient in competition assays [4]. In agreement with the competition assay, it was independently demonstrated that the same 128-aa. peptide efficiently binds nucleosome beads but not when the AT hook is mutated [13]. However, both studies concluded that the 128-aa. peptide contains residues outside the AT hook important for chromatin and nucleosome interaction. We attempted to address this in further detail, but we were unable to detect expression of a construct encoding the C-terminal 161 aa. of MEL-28 fused to GFP (S4B Fig; GFP::MEL-28^1624-1784^). A shorter 48-aa. fragment containing a single AT hook localized similarly to free GFP (S4A Fig GFP::MEL-28^1740-1784^). As a complementary approach, we examined the consequences of deleting the AT hooks from full-length MEL-28. We first compared *mel-28/+* embryos expressing GFP::MEL-28^1-1629^ (GFP::MEL-28^ΔAT^) with *mel-28* embryos expressing full-length MEL-28 fused to GFP. Time-lapse confocal microscopy demonstrated that the *mel-28/+*; GFP::MEL-28^1-1629^ embryos developed normally and the fluorescent protein localized similarly to GFP::MEL-28 (Fig 6A; compare left and middle panels; S9 and S10 Videos). In the absence of endogenous MEL-28, GFP::MEL-28^1-1629^ still accumulated at the periphery of interphase nuclei and to kinetochores of mitotic chromosomes (Fig 6A; right panels; S10 Video). This was in contrast to the severe phenotypes observed in MEL-28^loop2mut^::GFP embryos (Fig 3A) and suggested that MEL-28’s function in post-mitotic nuclear assembly is not strictly dependent on the AT hook domain. However, *mel-28*; GFP::MEL-28^1-1629^ embryos were unviable (Table 1) and displayed several defects. Most prominently, daughter nuclei were often (n = 5/7) trapped at the cleavage furrow during cytokinesis of the anterior AB blastomere of two-cell stage embryos (Fig 6A, right panels; 27:31–34:30). More direct evidence for chromosome segregation failure was obtained by immunofluorescence analysis of four-cell stage embryos, which also demonstrated that NPP-10C/NUP96 and other Nups accumulated at the NE of *mel-28*; GFP::MEL-28^1-1629^ embryos, albeit in an irregular pattern (Fig 6E). In addition, nuclear growth was significantly reduced in GFP::MEL-28^1-1629^ embryos (Fig 6A, third row; Fig 6B), consistent with defects in NPC-mediated nucleocytoplasmic transport [26]. While nuclei from *mel-28*; GFP::MEL-28 and *mel-28/+*; GFP::MEL-28^1-1629^ grew to the same size (363.8 ± 19 μm^3^ and 363.3 ± 63 μm^3^; respectively), the maximum volume of P1 nuclei was reduced by 32% in *mel-28*; GFP::MEL-28^1-1629^ embryos (346.6 ± 44 μm^3^). We also noticed that the nucleoplasmic pool of GFP::MEL-28^1-1629^ was strongly diminished in *mel-28* embryos compared to GFP::MEL-28 in *mel-28* embryos and GFP::MEL-28^1-1629^ in *mel-28/+* embryos (Fig 6A and 6C). Whereas the ratio between nucleoplasmic and cytoplasmic GFP signal was similar between *mel-28*; GFP::MEL-28 and *mel-28/+*; GFP::MEL-28^1-1629^ embryos (5.60 ± 1.29 and 4.72 ± 0.99; respectively), the ratio was 87% lower in *mel-28*; GFP::MEL-28^1-1629^ embryos (0.76 ± 0.18). These data are compatible with a model in which GFP::MEL-28^1-1629^ has reduced affinity for interphase chromatin and therefore accumulates at NPCs: in *mel-28/+* embryos interaction of GFP::MEL-28^1-1629^ with endogenous MEL-28 accumulates the former in the nucleoplasm, potentially interacting with chromatin.

During time-lapse recordings of 2-cell stage *mel-28* embryos, we realized that division of the P1 blastomere was much delayed relatively to the AB division. In wild-type embryos the P1 cell division is delayed by ~2.5 min compared to AB division. This P1 delay is dependent on checkpoint proteins and is thought to have evolved to protect the germ-line lineage from aneuploidy. Thus, inhibition of DNA replication or induction of DNA damage is typically associated with extended P1 delay. When we compared embryos expressing GFP::MEL-28^1-1629^ an increase in P1 delays by 176% was observed in *mel-28* versus m*el-28/+* embryos (423.5 ± 61.9 sec versus 154.1 ± 59.2 sec; Table 2; Fig 6D). The presence of chromatin bridges in *mel-28*; GFP::MEL-28^1-1629^ embryos (Fig 6E) suggested that chromosomes might be entangled, potentially as consequence of stalled replication and/or double-stranded DNA breaks. To address if the DNA damage checkpoint indeed is involved in the extended P1 delay in *mel-28*; GFP::MEL-28^1-1629^ embryos, we depleted ATL-1, the *C*. *elegans* homolog of ATR by RNAi [27]. This mitigated the P1 delay (285.7 ± 67.9 sec), which suggested that removal of the AT-hook domain from MEL-28 activates DNA damage and thereby an exaggerated delay of P1 cell division. However, depletion of ATL-1 did not fully rescue P1 cell-cycle timing, which suggests that other checkpoints are also activated in *mel-28*; GFP::MEL-28^1-1629^ embryos. In conclusion, although GFP::MEL-28^1-1629^ localizes properly to the NE and kinetochores, depletion of MEL-28’s AT-hook domain causes reduced nuclear growth, mis-segregation of chromosomes and activates the ATR DNA damage checkpoint.

**Table 2:** Loss of MEL-28’s AT Hooks causes checkpoint-dependent cell division delays.

Strain	n^a^	AB division^b^ (average +/- S.D.^c^)	P1 division^d^ (average +/- S.D.)	P1 delay^e^ (average +/- S.D.)	P1/AB ratio^f^ (average +/- S.D.)
*mel-28/+*; GFP::MEL-28^1-1629^; Control RNAi	15	828.6 +/- 33.1	982.7 +/- 81.4	154.1 +/- 59.2	1.2 +/-0.06
*mel-28*; GFP::MEL-28^1-1629^; Control RNAi	10	775.8 +/- 145.7	1199.3 +/- 151.2**	423.5 +/- 61.9***	1.6 +/-0.17***
*mel-28*; GFP::MEL-28^1-1629^; *atl-1 RNAi*	15	807.3 +/- 128.9	1093 +/- 133.2	285.7 +/- 67.9^ΨΨ^	1.4 +/-0.11^Ψ^

Significant differences by two-tailed t-test:

** different from control *mel-28*/+ embryos, p<0.01;

*** different from control *mel-28*/+ embryos, p<0.001;

^Ψ^ different from *mel-28* control RNAi embryos p<0.01;

^ΨΨ^ different from *mel-28* control RNAi embryos p<0.001.

^a^ Number of embryos analyzed via real-time DIC microscopy.

^b^ Time in seconds between P0 cytokinesis onset and AB cytokinesis onset.

^c^ Standard deviation.

^d^ Time in seconds between P0 cytokinesis onset and P1 cytokinesis onset.

^e^ Time in seconds between AB cytokinesis onset and P1 cytokinesis onset.

^f^ ratio of time to P1 over time to AB cytokinesis onset.

### MEL-28/ELYS localization domains are evolutionary conserved

**Fig 7:**
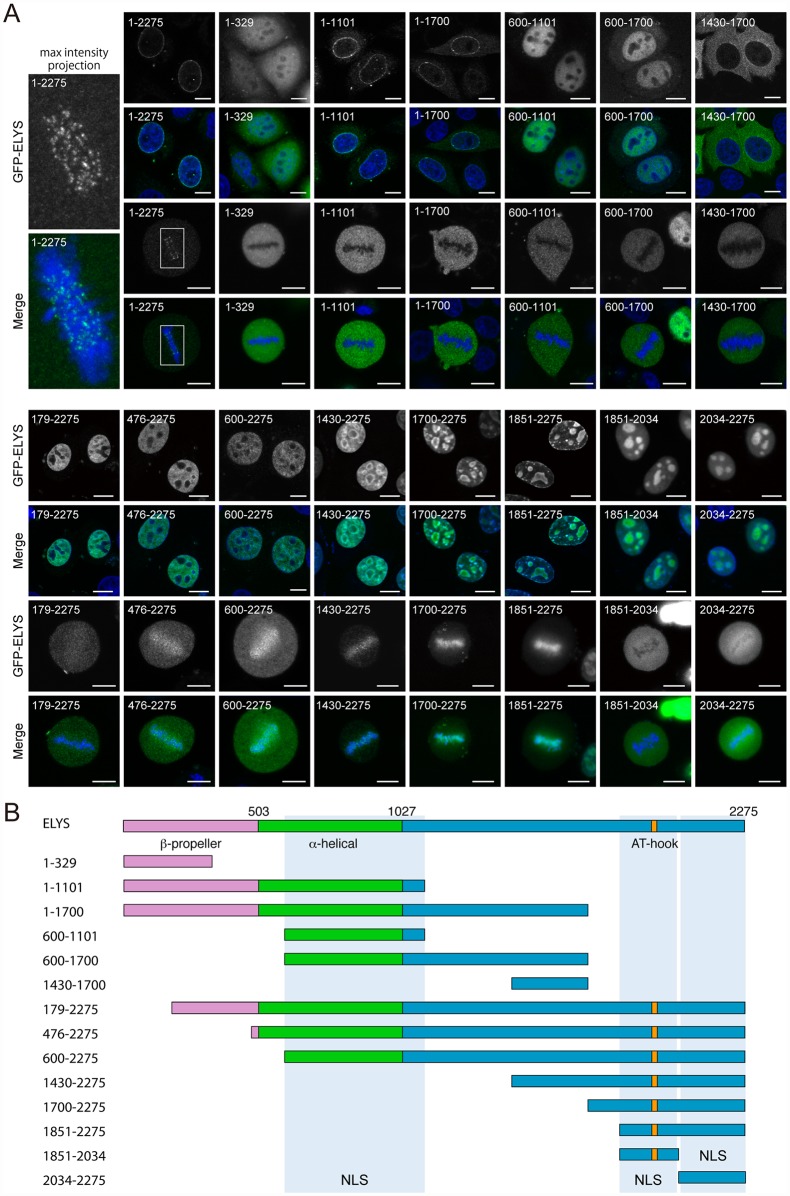
Mapping of ELYS localization domains. (A) Transiently transfected HeLa cells expressing full-length human ELYS (1–2275) or ELYS fragments fused to GFP (green in merge) were fixed and counterstained to visualize DNA (blue in merge). Maximum intensity projection of z-sections spanning the metaphase plate is shown for ELYS; other images represent single confocal sections. Scale bars, 10 μm. (B) Schematic representation of ELYS and analyzed fragments. Orange (aa. 1980–1989) boxes indicate AT-hook sequences. Truncations containing ELYS aa. residues 600–1101, 1851–2024 and/or residues 2034–2275 (blue shading) were efficiently imported.

To explore the degree of conservation of localization domains we expressed human full-length ELYS (ELYS^1-2275^) and 14 ELYS truncations fused to GFP in HeLa cells. As reported, ELYS^1-2275^ was enriched at the NE in interphase and in a pattern coincident with kinetochores in metaphase (Fig 7A; S5 Fig; punctate localization on metaphase chromosomes was observed in single confocal sections as well as in maximum intensity projections). Two fragments containing the entire β-propeller and α-helical domains (ELYS^1-1101^ and ELYS^1-1700^) still accumulated at the NE but had increased cytoplasmic signal, suggesting that, like for MEL-28, sequences outside the β-propeller and α-helical domains contribute to efficient NPC targeting (Fig 7A; S6 Fig). In contrast, all truncations from the N-terminal end abolished NE signal, including a deletion of ELYS aa. 1–178 (ELYS^179-2275^), indicating that the β-propeller is critically required for incorporation of ELYS into the NE. A short N-terminal fragment, ELYS^1-329^, was also not detected at the NE, which implies that although the first 178 aa. of ELYS are needed for NPC localization, they are not sufficient.

Two internal fragments, ELYS^600-1101^ and ELYS^600-1700^, were nuclear in interphase whereas ELYS^1430-1700^ was mostly cytoplasmic (Fig 7). This suggests that both MEL-28 (Fig 5; MEL-28^846-1071^) and ELYS have at least one NLS at equivalent locations within the central region of the protein. Nuclear accumulation was also observed for two non-overlapping C-terminal fragments, ELYS^1851-2034^ and ELYS^2034-2275^. In agreement with earlier predictions [5], this suggests the presence of NLS’s in the AT-hook-containing last 425 aa. of ELYS, similar to our mapping of a potential NLS to the AT-hook domain of MEL-28 (Fig 5; MEL-28^1188-1784^) and would represent another functional conservation between ELYS and MEL-28. We also noted that the shortest C-terminal ELYS fragments were enriched in nucleoli, whereas longer fragments (e.g. ELYS^179-2275^, ELYS^476-2275^, and ELYS^600-2275^) were excluded from these compartments (Fig 7).

Interestingly, all 14 ELYS truncations localized differently from full-length ELYS during metaphase. The three N-terminal fragments (ELYS^1-329^, ELYS^1-1101^, and ELYS^1-1700^) and the three internal fragments (ELYS^600-1101^, ELYS^600-1700^, and ELYS^1430-1700^) were not detected on mitotic chromosomes (Fig 7). In contrast, truncations from the N-terminal end increased the abundance of ELYS on chromosomes aligned on the metaphase plate. Importantly, the pattern was more diffuse on the chromosomes compared to the punctate pattern of full-length ELYS (S5 Fig). This was particularly prominent for ELYS^1700-2275^ and ELYS^1851-2275^, but was also observed for the longer ELYS^476-2275^, ELYS^600-2275^, and ELYS^1430-2275^ fragments. These results suggest that the C-terminus of ELYS has affinity for chromatin but that the ability to interact with chromosomes is reduced in the context of full-length ELYS, which specifically localizes to kinetochores. Thus, we conclude that association with mitotic chromosomes is also conserved from *C*. *elegans* to humans. Because of the similarity between MEL-28 and ELYS in terms of structural organization despite low primary sequence homology, we propose that the functional assignments for MEL-28 domains presented in this work are likely to be relevant in more complex animals, including humans.

## Discussion

**Fig 8:**
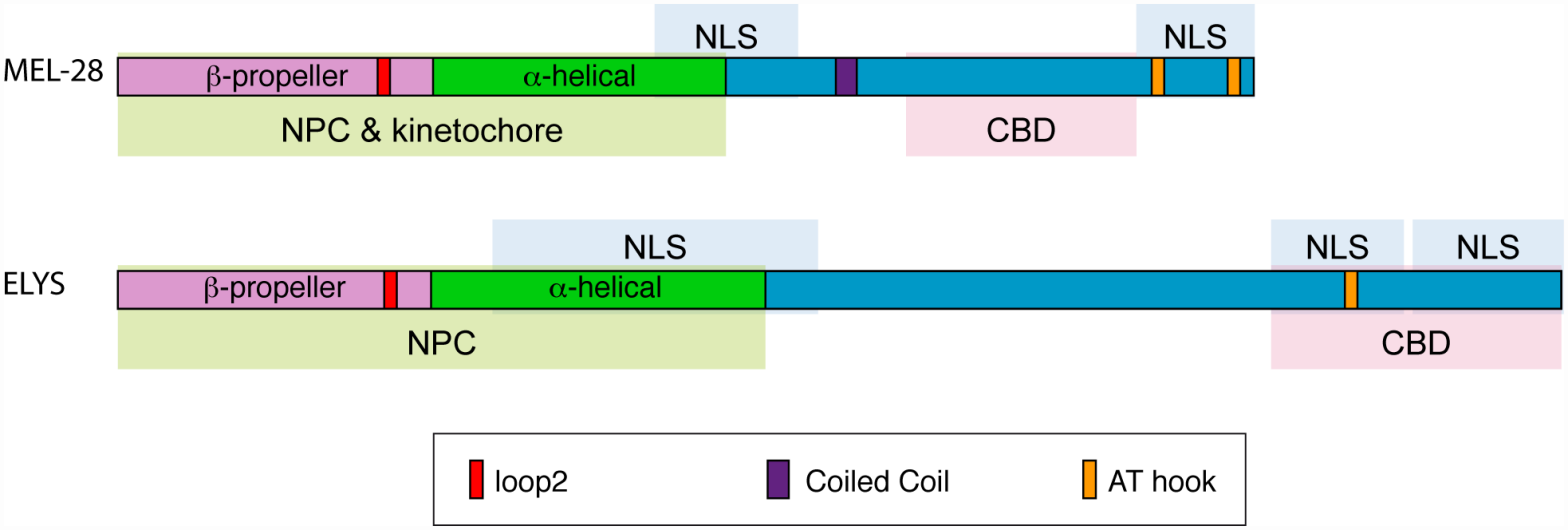
Overview of MEL-28 and ELYS localization domains. The N-terminal halves of MEL-28 and ELYS are sufficient to localize to NPCs (green shading) although less efficiently than full-length proteins. In the case of MEL-28, the N-terminus is also sufficient to localize to kinetochores. Both proteins contain central and C-terminal domains that are imported into nuclei (blue shading) and C-terminal domains that confer binding to chromatin (pink shading). A conserved loop2 motif in the N-terminal β-propeller is important for NPC localization in the context of truncated proteins. Both the loop2 motif and the AT-hook domain of MEL-28 are essential for embryonic viability.

*C*. *elegans* MEL-28 and human ELYS have divergent amino acid sequences, with at best 23% sequence identity [10]. In spite of this, we report that the functional domains of invertebrate and vertebrate orthologs are remarkably well conserved (Fig 8).

Previous work demonstrated that MEL-28/ELYS is essential for mitotic chromosome segregation in *C*. *elegans* and vertebrates [9, 10, 28]. Here we show that MEL-28 is also required for meiotic chromosome segregation in *C*. *elegans* oogenesis. In *C*. *elegans*, chromosome segregation during female meiosis is kinetochore-independent, and instead depends on microtubule growth in the region between separating chromosomes and lateral microtubule attachments to the separating chromosomes [20, 29]. It may be that these lateral attachments to chromosomes are less stable in the absence of MEL-28, leading to failure of chromosome segregation. Alternatively there could be defects to the architecture of the meiotic spindle when MEL-28 is disrupted, as has been shown for the mitotic spindle in *mel-28* RNAi-treated embryos [9, 21]. It is important to note that the cell cycle proceeds in *mel-28* embryos despite the penetrant failure in meiotic chromosome segregation, which suggests that *mel-28* does not affect the anaphase-promoting complex [30, 31].

In both *C*. *elegans* and HeLa cells, full-length MEL-28/ELYS localizes to the nucleoplasm and NPCs at interphase and to the kinetochore at mitosis [9, 10, 12, 28]. Here we observed that in *C*. *elegans*, localization to NPCs and the kinetochore is dependent on both the N-terminal β-propeller domain and the central α-helical domain, corresponding to the N-terminal 956 aa. residues. Mammalian ELYS NPC localization also requires the β-propeller and α-helical domains [15] and here we have shown that these domains are also necessary for the localization of ELYS to kinetochores at metaphase. Similar to previous studies of human ELYS [15], we have found that the conserved loop decorating blade 6 (“loop2”) is structurally conserved amongst the vertebrate and invertebrate MEL-28/ELYS homologs. When loop2 was disrupted by five substitution mutations in mouse ELYS, this prevented a 1018-aa. N-terminal ELYS fragment (corresponding to the β-propeller and α-helical domains) from localizing properly to the NE [15]. We found disruption of loop2 within an equivalent N-terminal fragment of MEL-28 (aa.1-956) caused a reduction of localization at the NPC and nucleoplasm, with a corresponding increase in cytoplasmic fluorescence. Interestingly, the full-length MEL-28 fusion with the loop2 defect had the wild-type localization pattern, suggesting that domains in the C terminus contribute to nuclear rim localization. Even so, mutations of loop2 severely disrupted MEL-28 function and caused cell cycle delay, nuclear expansion defects, problems with chromosome segregation during mitosis and meiosis, and ultimately embryonic inviability. However, NPC components were recruited to the reforming nuclei relatively efficiently. This suggests that the chromosomal functions of MEL-28 are more sensitive to defects to loop2 than the nuclear pore functions of MEL-28.

*In vitro* analyses studying the C-terminal domain of ELYS using *Xenopus* extracts have suggested that there are at least two domains, including the AT hook, required for chromatin binding [4, 11, 13]. Our results studying the C terminus of human ELYS are consistent with this. We identified at least two domains needed for metaphase chromatin localization. The C-terminal end of ELYS corresponding to aa. 1851–2275 bound to metaphase chromatin. However the aa. 1851–2034 fragment (which includes the AT hook) and a smaller aa. 2034–2275 C-terminal fragment were both excluded from metaphase chromatin, suggesting that both the AT hook and the domain C-terminal to the AT hook are required for metaphase chromatin binding.

The *C*. *elegans* MEL-28 data also suggest that both the AT hooks and other C-terminal domains are involved in chromatin binding. *C*. *elegans mel-28(t1684)* embryos expressing GFP::MEL-28^1-1629^ had reduced fluorescence in the nucleoplasm at interphase, consistent with an inefficient chromatin binding. These embryos also showed defects in recruitment of NPC components that would be expected if MEL-28 could not effectively bind to chromatin [3, 4]. We studied multiple C-terminal fragments of MEL-28 (that also lacked the N-terminal β-propeller and the central α-helical domains). Such fragments that include aa. 1239–1601 localized to the metaphase chromatin, but fragments lacking this domain were excluded from metaphase chromatin. This suggests that aa. 1239–1601, just N-terminal to the AT hooks in MEL-28, comprise a chromatin-binding domain. Notably, MEL-28 fragments with an intact N terminus (including the β-propeller domain and the central α-helical domain) localized to the kinetochore regardless of the presence of aa. 1239–1601, showing that metaphase kinetochore localization does not require this domain. With human ELYS, in contrast, fragments were completely excluded from the chromatin and kinetochores unless they contained the C terminal domain including aa. 1851–2275.

In contrast to the behavior of C-terminal MEL-28 and ELYS fragments, full-length *C*. *elegans* and human proteins were enriched at kinetochores with no apparent affinity for other parts of the metaphase chromosomes. Moreover, disruption of kinetochores blocks recruitment of MEL-28 to mitotic chromosomes [9]. However, several observations indicate that full-length MEL-28 and ELYS also interact with chromatin. Firstly, ELYS bound to chromatin in interphase *Xenopus* egg extracts [4, 11, 13]. Secondly, DamID experiments in *C*. *elegans* adults showed specific interaction of MEL-28 throughout all chromosomes [32]. As a possible explanation for the different behavior at interphase and mitosis we speculate that MEL-28 and ELYS might undergo conformational changes in mitosis that lower their affinity for chromatin. Upon deletion of N-terminal regions, the chromatin association domain(s) in the C-terminus of MEL-28 and ELYS become more accessible and confer binding to metaphase chromosomes. Such a “shielding” mechanism is concordant with the gradual increase in association to metaphase chromosomes as more residues are deleted from the N-terminus of ELYS. Alternatively, or in combination with conformational changes of MEL-28 and ELYS, condensed mitotic chromosomes might provide a less favorable binding site for MEL-28/ELYS.

MEL-28 is efficiently targeted to the NPC and the kinetochore even without AT hooks. However, the ΔAT-hooks version of MEL-28 clearly lacks MEL-28 function; *mel-28(t1684)* embryos expressing MEL-28^1-1629^ were defective in NPC assembly and nearly all died before hatching. This shows that having MEL-28 placed at the NE is not sufficient for efficient recruitment of the remaining components of the NPC but that this depends on the AT-hook domain. In addition, these embryos show chromatin bridges and activate a checkpoint associated with DNA breakage. Previous work has suggested a role for MEL-28 in chromosome congression and segregation [9, 10], and our observations suggest that these functions require the AT hooks.

The second, or most C-terminal, of the two predicted AT hooks clustered at the C terminus is a canonical AT hook whereas the penultimate is less well conserved [10]. Interestingly, the MEL-28 fusion missing its last AT hook retained some MEL-28 function, as *mel-28(t1684)* animals expressing this fusion showed partial penetrance embryonic lethality, with over one third of the embryos surviving (Table 1). Since removal of both AT hooks causes 99% embryonic lethality, either the penultimate AT hook or the short domain between the AT hooks must contribute to MEL-28 function. In either case, most *mel-28(t1684)* embryos expressing the version lacking the last AT hook are unviable, so the last AT hook is clearly needed for full MEL-28 function.

In conclusion, human ELYS and *C*. *elegans* MEL-28 have similar functional domains. Both orthologs depend on an intact β-propeller domain and central α-helical domains for NPC and kinetochore organization. The β-propeller domain contains several loops, and our work has demonstrated that loop2, a region that contributes to ELYS localization in mammals [15], is also critical for MEL-28 function. Both MEL-28 and ELYS also have several putative NLS’s traversing the central and C terminal regions of the protein and a C-terminal chromatin-binding domain. One major difference between MEL-28 and ELYS is that chromatin and kinetochore binding is strictly dependent on the C-terminal chromatin-binding domain in ELYS. In contrast, MEL-28 fragments lacking the C terminus are still delivered to the kinetochore as long as the N terminus is intact although in a more irregular manner. It is possible that MEL-28 kinetochore localization is more robust to perturbation because of the unique holocentric structure of the kinetochore in *C*. *elegans*.

## Materials and Methods

### Plasmid constructions

DNA fragments to express MEL-28 full length and truncations were generated by PCR amplification (KAPA HiFi; KAPA Biosystems, Wilmington, USA) or restriction enzyme digestion and inserted into appropriate cloning vectors. In all cases, *mel-28* introns were maintained. Plasmid details are listed in S1 Table.

To construct GFP-human ELYS (NCBI accession number: NP_056261.4), total RNAs from HeLa, K562 and WI-38 cells were isolated by FastPure RNA kit (TaKaRa Bio Inc., Shiga, Japan), and then cDNAs were generated by using SuperScript III First-Strand synthesis system (Invitrogen, Waltham, MA) according to manufacturer’s protocol. The coding region of ELYS was PCR-amplified using primers listed in S2 Table and inserted into the pEGFP-C1 vector (Clontech Laboratories, Palo Alto, CA) at the *Xho*I site by In-Fusion reaction (Clontech). Other ELYS fragments were amplified by PCR using the plasmid harboring full-length ELYS as a template and inserted into the pEGFP-C1 vector as describe above. DNA sequencing of all ELYS fusion plasmids was outsourced to the TaKaRa Bio Inc. Compared to the database sequence, 5 out of 5, 6 out of 7 and 2 out of 2 clones from HeLa, K562 and WI-38 cells, respectively, contained a mutation from A to G at position 2648, resulting in an amino acid substitution from N to S at the position 883. Since the mutation was predominant in three different cell lines, we decided to use this ELYS sequence in this report.

### Nematode strains and transgenesis

The wild type strain used was the *C*. *elegans* Bristol strain N2. Transgenic strains were generated by any of three different methods: MosSCI [33], CRISPR-Cas9 [17] or microparticle bombardment [34]. GE2633 (*mel-28(t1684)*) was obtained from the *Caenorhabditis* Genetic Centers. Other strains are listed in S3 Table. Strains were cultured at 15–25°C using standard *C*. *elegans* methods [35].

### *C*. *elegans* embryonic lethality rescue experiments

Rescue experiments were performed according to the promoter used to express the different MEL-28 fragments. For constitutive promoters homozygous L4 larvae were placed on individual plates to develop and lay eggs for 24 h at 20°C. Then, the adults were removed and the number of eggs was determined. Twenty four hours later embryonic lethality was calculated by counting unhatched embryos. For constructs with the *hsp-16*.*41* heat shock inducible promoter, young gravid adults were incubated for 1 h at 32°C and allowed to recover and lay eggs for 24 h at 20°C. The adults were then removed and rescue of embryonic lethality was determined by the presence of viable offspring after 24 h at 20°C.

### *C*. *elegans* RNAi

We carried out RNAi as described [36] with minor adaptations. In total, 10–15 synchronized L4 hermaphrodites were placed on NGM plates (+ 1 mM IPTG + 100 μg/ml ampicillin) seeded with *E*. *coli* producing double-stranded RNA (*alt-1* RNAi clone sjj_T06E4.3 from [37]) and incubated for 20-24h at 20°C before analysis of cell cycle timing by live DIC microscopy.

### Cell culture

HeLa cells were a gift from Dr. Hiroshi Kimura (see [38] for the cell origin). WI-38 cells were purchased from ATCC (Manassas, VA, USA). These cells were maintained in DME medium containing 10% fetal bovine serum (FBS) at 37°C in a humidified 5% CO_2_. K562 cells were obtained from the Riken Cell Bank (Tsukuba, Japan) and maintained in RPMI1640 medium containing 10% FBS. HeLa cells were grown in a glass-bottom culture dish (MatTech, USA). GFP fusion plasmids (1 μg) were transfected into the cells with Lipofectamine 2000 (Invitrogen) according to manufacturer’s protocol. After 24 hours transfection, the cells were fixed with 4% formaldehyde for 10 min, permeabilized with 0.1% Triton X-100 in PBS for 5 min. For immunostaining, the cells were blocked by blocking buffer (PBS containing 10% Blocking One (Nacalai tesque, Japan) and 0.1% Triton X-100), and then probed with anti-CENP-A antibody (generous gift from Dr. Tatsuo Fukagawa (Osaka University), [39]), followed by Alexa Fluor 568-conjugated anti-mouse IgG secondary antibody (1:500, Lifetechnologies, USA). The cells were stained with 4′,6-diamidino-2-phenylindole (DAPI) at 100 ng/ml for 10 min at room temperature. After washing 3-times with 0.1% Triton X-100 in PBS, the cells were mounted on ProLong Diamond antifade mountant (Molecular Probes, Carlsbad, CA). The cells were observed by confocal microscopy (LSM510META and LSM780; Zeiss; operated by built-in software) equipped with a C-Apo 40x NA 1.2 water immersion lens.

### *C*. *elegans* immunofluorescence

*C*. *elegans* embryos and larvae were collected and processed by freeze cracking and methanol fixation as described [40]. The following primary antibodies were used: mouse monoclonal antibody (mAb) 414 (Covance, Princeton, NJ, USA,1:250), mouse monoclonal antibody MH27 (1:50; [41], provided by the Developmental Studies Hybridoma Bank), rabbit polyclonal α-HCP-3 antiserum MH3N (1:200; generous gift from Dr. Mark Roth [42]), rabbit polyclonal α-NPP10-C/NUP96 antiserum GBLC (1:300; [21]), rabbit polyclonal α-MEL-28 antiserum BUD3 (1:200–250; [10]). Secondary antibodies were Alexa Fluor 546-conjugated goat anti-mouse antibodies (Invitrogen, 1:1000), Alexa Fluor 488- and Alexa Fluor 633-conjugated goat anti-rabbit antibodies (Invitrogen, 1:1000). For DNA staining, Hoechst 33258 (Hoechst) was used at 5 μg/ml. Confocal images for S1A Fig were obtained with a Nikon A1R microscope through a Plan Apo VC 60x/1.4 objective (Nikon, Tokyo, Japan) using a pinhole of 1 airy unit. All other immunofluorescence images were acquired with a confocal Leica SPE microscope equipped with an ACS APO 636/ 1.3 objective (Leica, Wetzlar, Germany) using a pinhole of 1 airy unit.

### Live imaging

*C*. *elegans* samples were mounted between a coverslip and a 2% agarose pad; embryos were released by dissecting young adult hermaphrodites and mounted in 3 μL M9 buffer, whereas larvae and adults were mounted in 3 μL 10 mM levamisole HCl (Sigma-Aldrich, St. Louis, MI, USA). For *in utero* imaging of oocytes and newly fertilized embryos, young adult hermaphrodites were anesthetized in 20 μL 5 mM ethyl 3-aminobenzoate methanesulfonate (aka Tricaine; Sigma-Aldrich), 0.5 mM levamisole HCl, 0.5x M9 for 15–20 minutes prior to mounting in 3 μL of the same buffer on 2% agarose pads. Vaseline was added between the slide and the coverslip to avoid compression of the animals and melted VALAP (1:1:1 mixture of Vaseline, lanolin, and paraffin) was used to seal the cover slip. Confocal epifluorescence and DIC images were recorded at 22–24°C with a Nikon A1R microscope through a Plan Apo VC 60x/1.4 objective (Nikon, Tokyo, Japan) using a pinhole of 1.2–1.4 airy unit.

### Image processing and analysis

For preparation of Fig panels images were processed with FIJI (fiji.sc/Fiji) and Adobe Photoshop CS5 or CS6 (Adobe, San Jose, CA, USA). Identical adjustment of brightness and contrast was applied to all comparable panels within each Fig without changing gamma. Quantification of fluorescence signal at the NE, cytoplasm and nucleoplasm was performed on raw 12 bit images. Fluorescence intensity was normalized by background subtraction; for *C*. *elegans*, images of wild type embryos acquired with identical microscope settings were used, with exception of S2B Fig

### Statistical analysis

Statistical analysis was performed with Origin 8.0 (OriginLab, Northampton, MA, USA), Microsoft Excel (Microsoft, Redmond, WA, USA) and online Graphpad tools (http://graphpad.com).

## Supporting Information

S1 Fig MEL-28 is ubiquitously expressed. (A) Embryos were fixed and analyzed with antibodies against MEL-28 and Hoechst to stain DNA (green and magenta in merge, respectively). Single confocal mid sections and maximum projections indicate that MEL-28 is uniformly expressed in all embryonic cells. Approximate developmental time is indicated from fertilization. (B) Maximum projections of confocal sections of L4 larva analyzed with Hoechst (blue in merge) and anti-MEL-28 and MH27 antibodies (green and red, respectively). (C) Maximum projection of confocal sections of adult and embryo showing ubiquitous GFP::MEL-28 expression in GFP knock-in strain. Insert represents a confocal mid section of L2 and L3 larvae. Arrow points to a mature oocyte with MEL-28 localization to condensed chromosomes. Scale bars, 10 μm.
(TIF)

S2 Fig Analysis of MEL-28 expression levels. (A) Compared to a strain that expresses GFP::MEL-28 from the endogenous *mel-28* locus after CRISPR/Cas9-mediated GFP knock-in (top panel), expression of GFP::MEL-28 full-length and mutant proteins from transgenes inserted by microparticle bombardment or MosSCI is either similar or lower, thus arguing against the possibility of artifacts induced by overexpression. Confocal images were acquired with identical settings (laser power = 7% and PMT high voltage = 150) except P_*mex-5*_::GFP::MEL-28 (yellow asterisk; laser power = 9%) and *mel-28*; P_*pie-1*_::GFP::MEL-28^Δ498–956^ (red asterisk; laser power = 8%). (B) Comparison of GFP::MEL-28 fragments expressed from heat shock-induced single copy transgenes containing the *hsp-16*.*41* promoter. Older embryos are shown because induction is inefficient in young embryos. Confocal images were taken with identical settings (laser power = 5% and PMT high voltage = 150). (C) A GFP::MEL-28^1740-1784^ fragment expressed under control of the *pie-1* promoter is visible in early embryos and localizes diffusely throughout the cell (laser power = 8% and PMT high voltage = 160). Wild type embryos not expressing GFP were observed with identical microscope settings and included as controls in A-C. Scale bars, 10 μm.
(TIF)

S3 Fig Impaired nuclear import and NPC localization of MEL-28^1-956_loop2mut^. (A) Confocal images of embryos expressing GFP::MEL-28 or MEL-28^1-956_loop2m^::GFP. Both embryos also expressed endogenous untagged MEL-28. Scale bars, 5 μm. (B) In interphase, the ratio of nucleoplasmic versus cytoplasmic GFP signal was ~4.4-fold higher for full-length MEL-28 compared to MEL-28^1-956_loop2m^ (3.41 ± 1.28 versus 0.77 ± 0.07). Mutation of MEL-28 loop2 (MEL-28^loop2m^::GFP) in the context of full-length protein did not reduce nuclear enrichment (3.97 ± 0.44), suggesting that the impaired import of MEL-28^1-956_loop2m^::GFP was mainly due to deletion of the C-terminal domain. (C) Accumulation of MEL-28^1-956_loop2m^::GFP at the NE (relative to kinetochore localization) was also specifically reduced (0.94 ± 0.09, 0.94 ± 0.1, and 0.14 ± 0.09, respectively). *** p<0.001 by unpaired two-tailed t-test.
(TIF)

S4 Fig Analysis of additional MEL-28 fragments. (A) Cropped images from embryos expressing different MEL-28 truncations fused to GFP. Except GFP::MEL-28 all embryos also expressed untagged endogenous MEL-28. (B) MEL-28 truncations for which several transgenic lines were obtained but without showing GFP expression, potentially reflecting reduced mRNA or protein stability.
(TIF)

S5 Fig Full-length ELYS, but not ELYS fragments, strongly accumulates at kinetochores at mitosis. Cells expressing full-length or truncated GFP-ELYS (green in merge) were analyzed by immunofluorescence with a specific antibody against kinetochore protein CENP-A (red in merge) and DAPI (blue in merge). Single confocal sections (A) and maximum projection images (B) of metaphase cells are shown. Full-length ELYS co-localizes extensively with CENP-A whereas several C-terminal fragments are diffusely associated with metaphase chromosomes. Scale bars, 10 μm.
(TIF)

S6 Fig The C-terminal domain of ELYS is required for efficient targeting to the nuclear envelope. Fluorescence intensity of the NE and cytoplasm was determined for HeLa cells transiently expressing GFP fused to full-length ELYS (ELYS^1-2275^), ELYS^1-1101^, or ELYS^1-1700^. The ratio of NE versus cytoplasmic fluorescence was reduced by 70–71% for the two truncated ELYS proteins. *** p<0.001 by unpaired two-tailed t-test.
(TIF)

S1 Video Maturing *C*. *elegans* oocytes expressing GFP::MEL-28 (green) and mCherry::HIS-58 (magenta) observed by confocal microscopy. Time is indicated relative to germinal vesicle breakdown. The video is a merge of two separate recordings: Frames -30 min to 2 min correspond to Fig 1A whereas frames 4 min to 44 min correspond to Fig 1B. Playback speed is 360-720x.
(AVI)

S2 Video Fertilized *C*. *elegans* oocytes expressing GFP::TBB-2 (green) and mCherry::HIS-58 (magenta) observed by confocal microscopy. Time is indicated relative to germinal vesicle breakdown. Corresponds to heterozygous *mel-28/+* (top) and homozygous *mel-28* (bottom) mutants in Fig 1C. Playback speed is 360x.
(AVI)

S3 Video Recording of early *C*. *elegans* embryos to evaluate MEL-28 coiled-coil domain. In the top, control embryo expressing GFP::MEL-28 and in the bottom, embryo expressing GFP::MEL-28^Δ1140–1186^ both observed by confocal microscopy. Corresponds to Fig 2C. Playback speed is 60x. Frames were taken every 3 seconds for 24 minutes.
(AVI)

S4 Video Recording of early *C*. *elegans* embryos to evaluate MEL-28 loop2 region. Heterozygous (top) and homozygous (bottom) *mel-28* embryos expressing MEL-28^loop2mut^::GFP observed by confocal microscopy. Corresponds to Fig 3A. Playback speed is 60x. Stacks of 7 focal planes were acquired every 15 seconds for 35 minutes; videos represent maximum projection images.
(AVI)

S5 Video Recording of early *C*. *elegans* embryo to evaluate last MEL-28 AT-hook motif. Embryo expressing GFP::MEL-28^1-1744^ observed by confocal microscopy. Corresponds to Fig 5. Playback speed is 60x. Frames were taken every 4.6 seconds for 34 minutes.
(AVI)

S6 Video Recording of gastrulating *C*. *elegans* embryos to evaluate MEL-28 chromatin binding domain. In the top, control embryo expressing GFP::MEL-28 and in the bottom, embryo expressing GFP::MEL-28^Δ1239–1728^ observed by confocal microscopy. Red and yellow arrowheads indicate examples of dividing cells. GFP::MEL-28 is associated with kinetochores and chromatin at metaphase and anaphase, whereas GFP::MEL-28^Δ1239–1728^ is absent from anaphase chromosomes. Corresponds to Fig 5. Playback speed is 60x. Frames were taken every 6.6 and 4.6 seconds, respectively, for 7.5 minutes.
(AVI)

S7 Video Recording of early *C*. *elegans* embryos to evaluate N-terminal vs. full-length MEL-28. Embryos expressing full-length MEL-28^loop2mut^::GFP (top) or MEL-28^1-956_l2m^::GFP (bottom) observed by confocal microscopy. Corresponds to Fig 5. Playback speed is 60x. Frames were taken every 6.6 and 4.6 seconds, respectively, for 3 minutes.
(AVI)

S8 Video Recording of gastrulating *C*. *elegans* embryos to evaluate minimal MEL-28 chromatin binding domain. Embryos expressing GFP::MEL-28^846-1350^ (top) or GFP::MEL-28^846-1601^ (bottom) observed by confocal microscopy. Corresponds to Fig 5. Playback speed is 60x. Frames were taken every 4.6 seconds for 6 minutes.
(AVI)

S9 Video Early *C*. *elegans* embryo expressing GFP::MEL-28 observed by confocal microscopy. Corresponds to Fig 6A. Playback speed is 60x. Frames were taken every 3 seconds for 32 minutes.
(AVI)

S10 Video Recording of early *C*. *elegans* embryos to evaluate MEL-28 AT-hook domain. Heterozygous (top) and homozygous (bottom) *mel-28* embryos expressing GFP::MEL-28^1-1629^ observed by confocal microscopy. Corresponds to Fig 6A. Playback speed is 60x. Frames were taken every 3 seconds for 32 minutes.
(AVI)

S1 Table Plasmids used in this study. (XLSX)

S2 Table Primers used in this study. (XLSX)

S3 Table Strains used in this study. (XLSX)
