# Supplementary figures and images for: Identification of Conserved MEL-28/ELYS Domains with Essential Roles in Nuclear Assembly and Chromosome Segregation
Source: PLoS Genet. 2016 Jun 24;12(6):e1006131. doi: 10.1371/journal.pgen.1006131 (PMC4920428; doi:10.1371/journal.pgen.1006131)

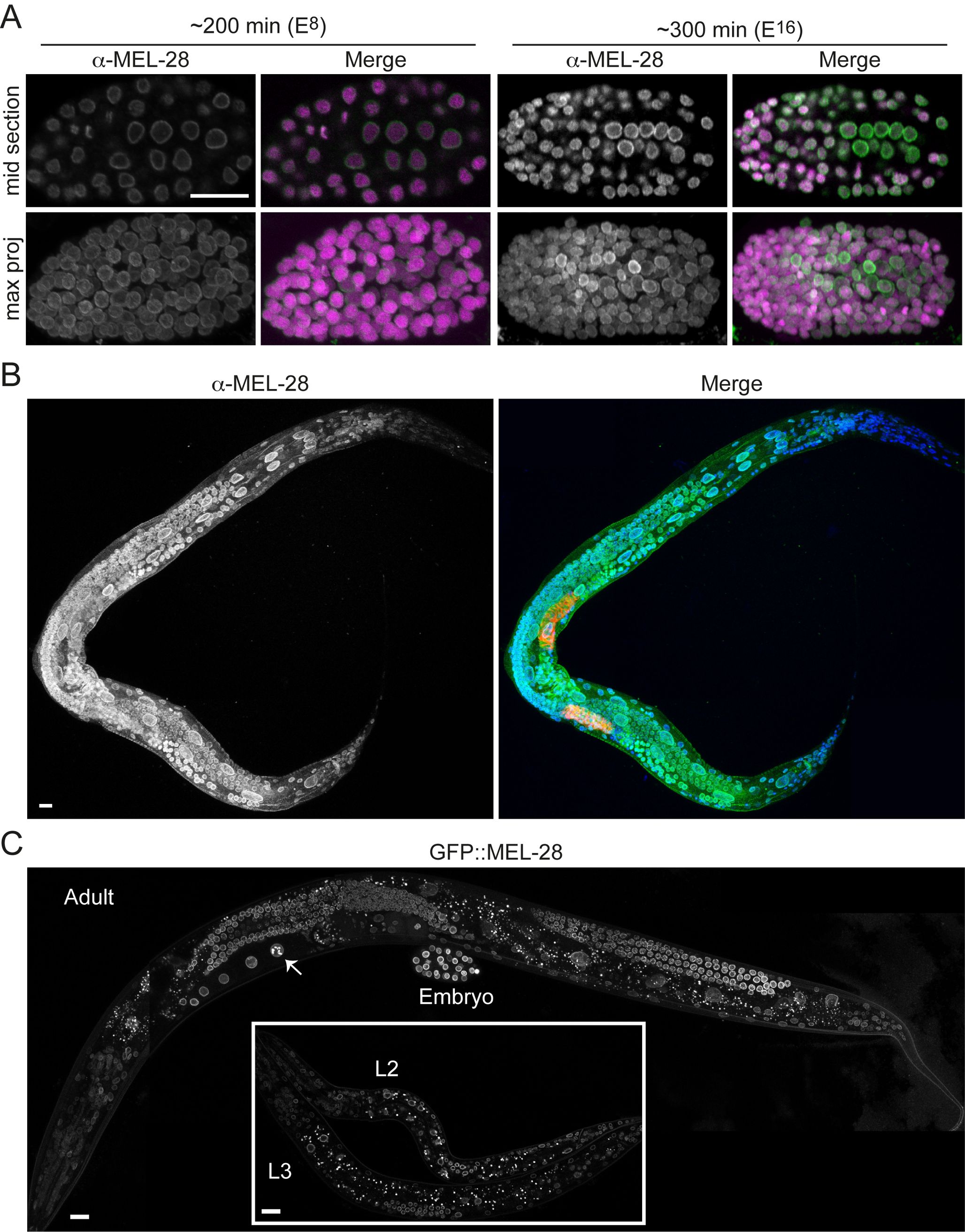

Supplement: S1 Fig — (A) Embryos were fixed and analyzed with antibodies against MEL-28 and Hoechst to stain DNA (green and magenta in merge, respectively). Single confocal mid sections and maximum projections indicate that MEL-28 is uniformly expressed in all embryonic cells. Approximate developmental time is indicated from fertilization. (B) Maximum projections of confocal sections of L4 larva analyzed with Hoechst (blue in merge) and anti-MEL-28 and MH27 antibodies (green and red, respectively). (C) Maximum projection of confocal sections of adult and embryo showing ubiquitous GFP::MEL-28 expression in GFP knock-in strain. Insert represents a confocal mid section of L2 and L3 larvae. Arrow points to a mature oocyte with MEL-28 localization to condensed chromosomes. Scale bars, 10 μm. (TIF) [file pgen.1006131.s001.tif]

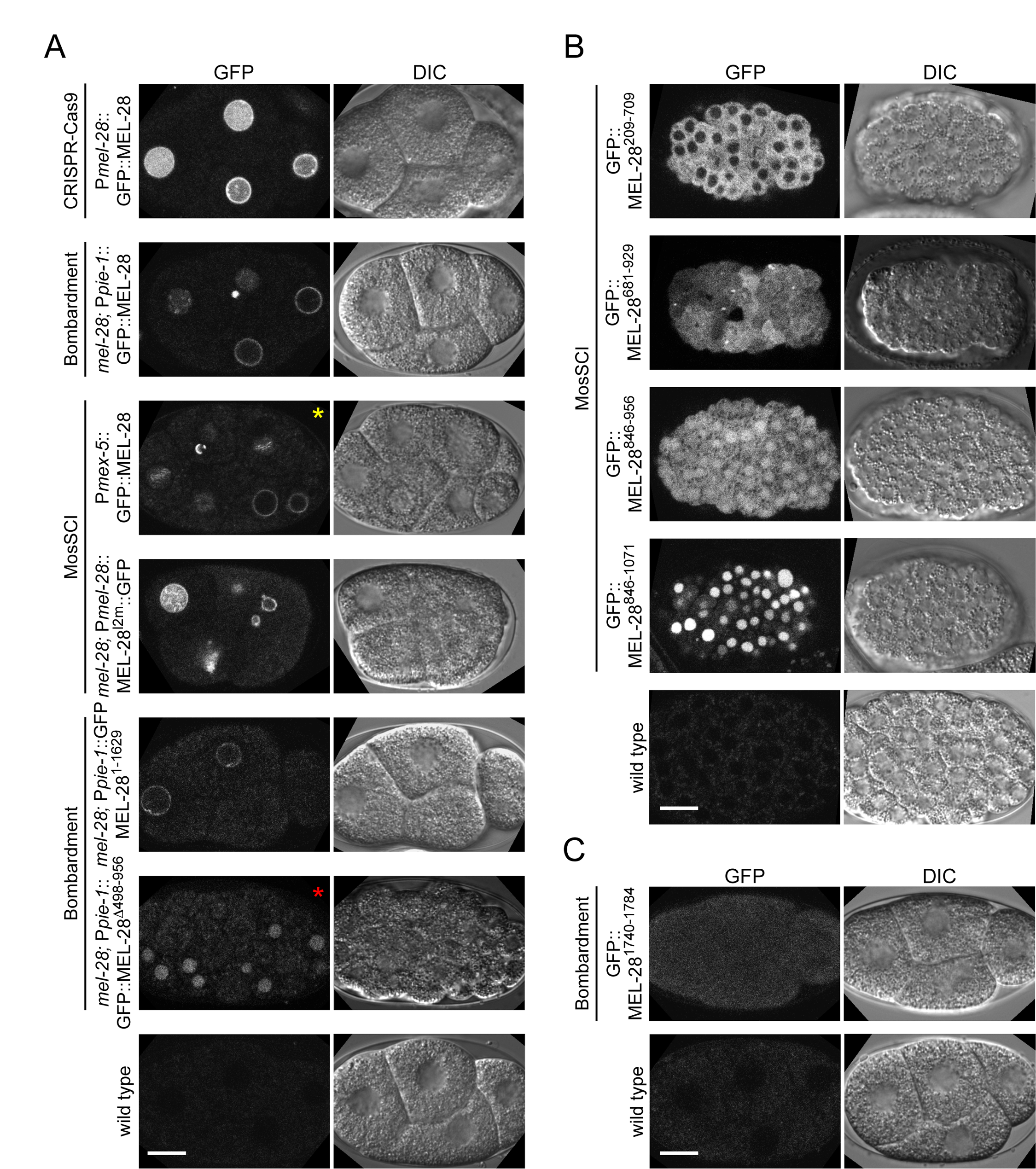

Supplement: S2 Fig — (A) Compared to a strain that expresses GFP::MEL-28 from the endogenous mel-28 locus after CRISPR/Cas9-mediated GFP knock-in (top panel), expression of GFP::MEL-28 full-length and mutant proteins from transgenes inserted by microparticle bombardment or MosSCI is either similar or lower, thus arguing against the possibility of artifacts induced by overexpression. Confocal images were acquired with identical settings (laser power = 7% and PMT high voltage = 150) except Pmex-5::GFP::MEL-28 (yellow asterisk; laser power = 9%) and mel-28; Ppie-1::GFP::MEL-28Δ498–956 (red asterisk; laser power = 8%). (B) Comparison of GFP::MEL-28 fragments expressed from heat shock-induced single copy transgenes containing the hsp-16.41 promoter. Older embryos are shown because induction is inefficient in young embryos. Confocal images were taken with identical settings (laser power = 5% and PMT high voltage = 150). (C) A GFP::MEL-281740-1784 fragment expressed under control of the pie-1 promoter is visible in early embryos and localizes diffusely throughout the cell (laser power = 8% and PMT high voltage = 160). Wild type embryos not expressing GFP were observed with identical microscope settings and included as controls in A-C. Scale bars, 10 μm. (TIF) [file pgen.1006131.s002.tif]

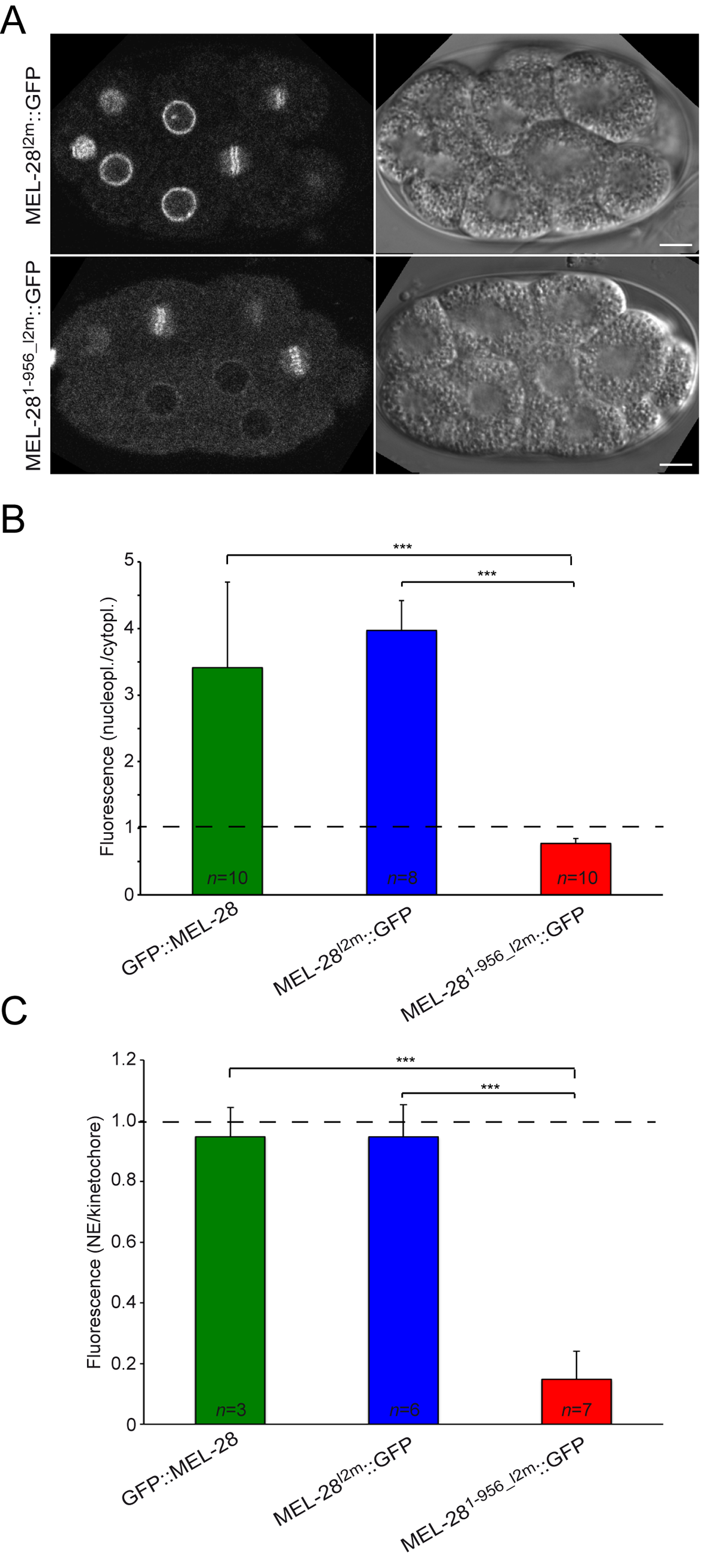

Supplement: S3 Fig — (A) Confocal images of embryos expressing GFP::MEL-28 or MEL-281-956_loop2m::GFP. Both embryos also expressed endogenous untagged MEL-28. Scale bars, 5 μm. (B) In interphase, the ratio of nucleoplasmic versus cytoplasmic GFP signal was ~4.4-fold higher for full-length MEL-28 compared to MEL-281-956_loop2m (3.41 ± 1.28 versus 0.77 ± 0.07). Mutation of MEL-28 loop2 (MEL-28loop2m::GFP) in the context of full-length protein did not reduce nuclear enrichment (3.97 ± 0.44), suggesting that the impaired import of MEL-281-956_loop2m::GFP was mainly due to deletion of the C-terminal domain. (C) Accumulation of MEL-281-956_loop2m::GFP at the NE (relative to kinetochore localization) was also specifically reduced (0.94 ± 0.09, 0.94 ± 0.1, and 0.14 ± 0.09, respectively). *** p<0.001 by unpaired two-tailed t-test. (TIF) [file pgen.1006131.s003.tif]

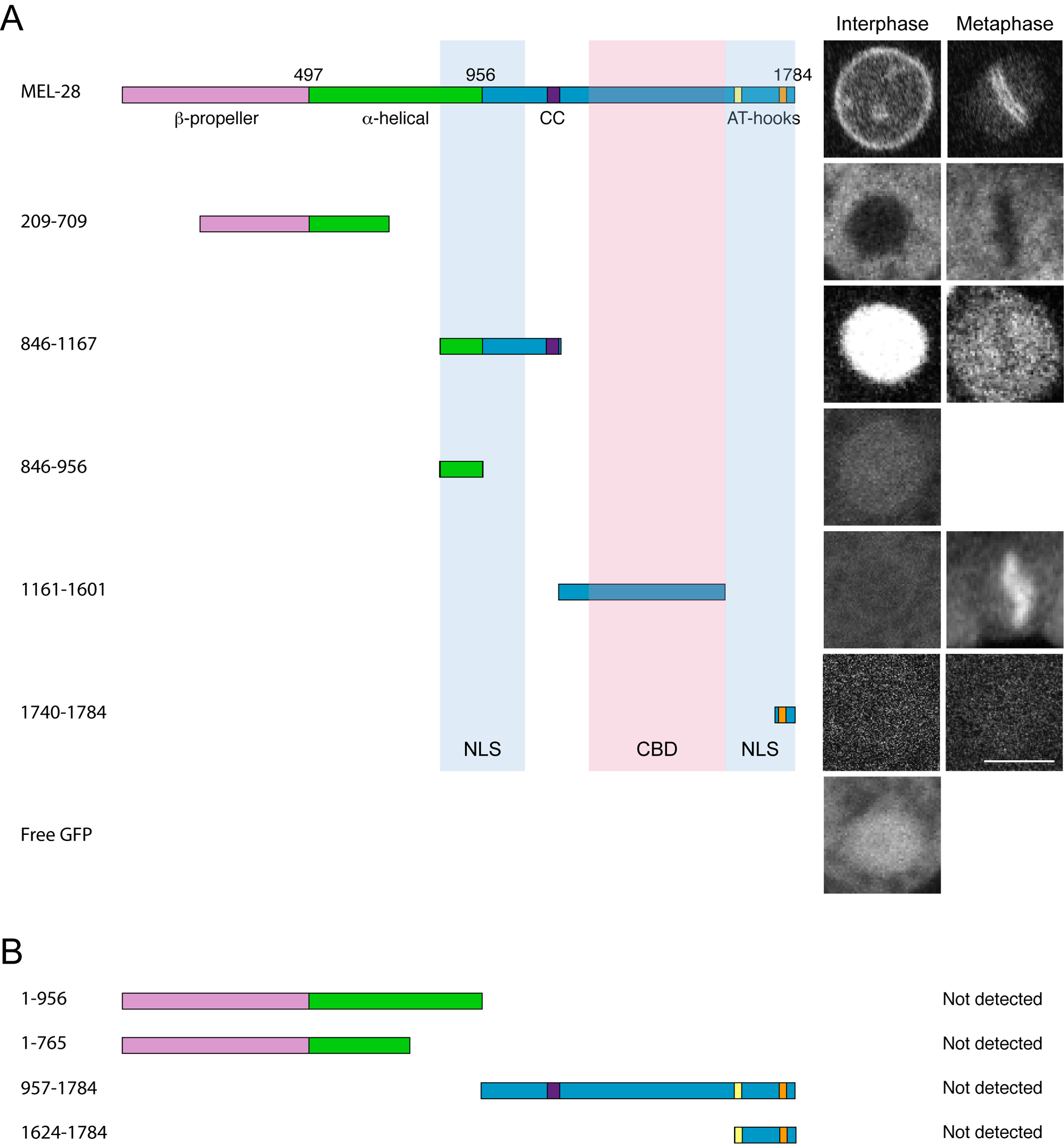

Supplement: S4 Fig — (A) Cropped images from embryos expressing different MEL-28 truncations fused to GFP. Except GFP::MEL-28 all embryos also expressed untagged endogenous MEL-28. (B) MEL-28 truncations for which several transgenic lines were obtained but without showing GFP expression, potentially reflecting reduced mRNA or protein stability. (TIF) [file pgen.1006131.s004.tif]

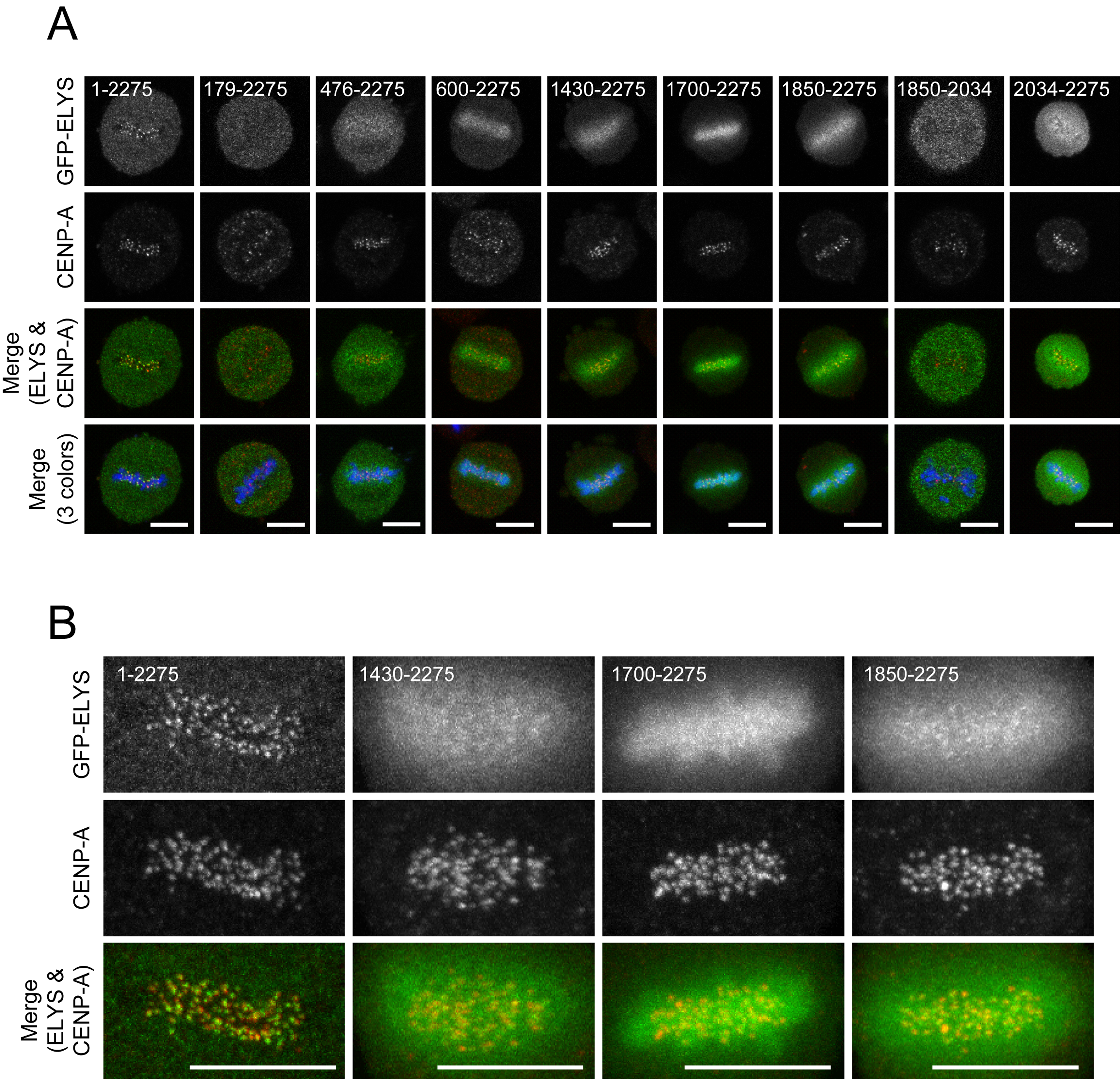

Supplement: S5 Fig — Cells expressing full-length or truncated GFP-ELYS (green in merge) were analyzed by immunofluorescence with a specific antibody against kinetochore protein CENP-A (red in merge) and DAPI (blue in merge). Single confocal sections (A) and maximum projection images (B) of metaphase cells are shown. Full-length ELYS co-localizes extensively with CENP-A whereas several C-terminal fragments are diffusely associated with metaphase chromosomes. Scale bars, 10 μm. (TIF) [file pgen.1006131.s005.tif]

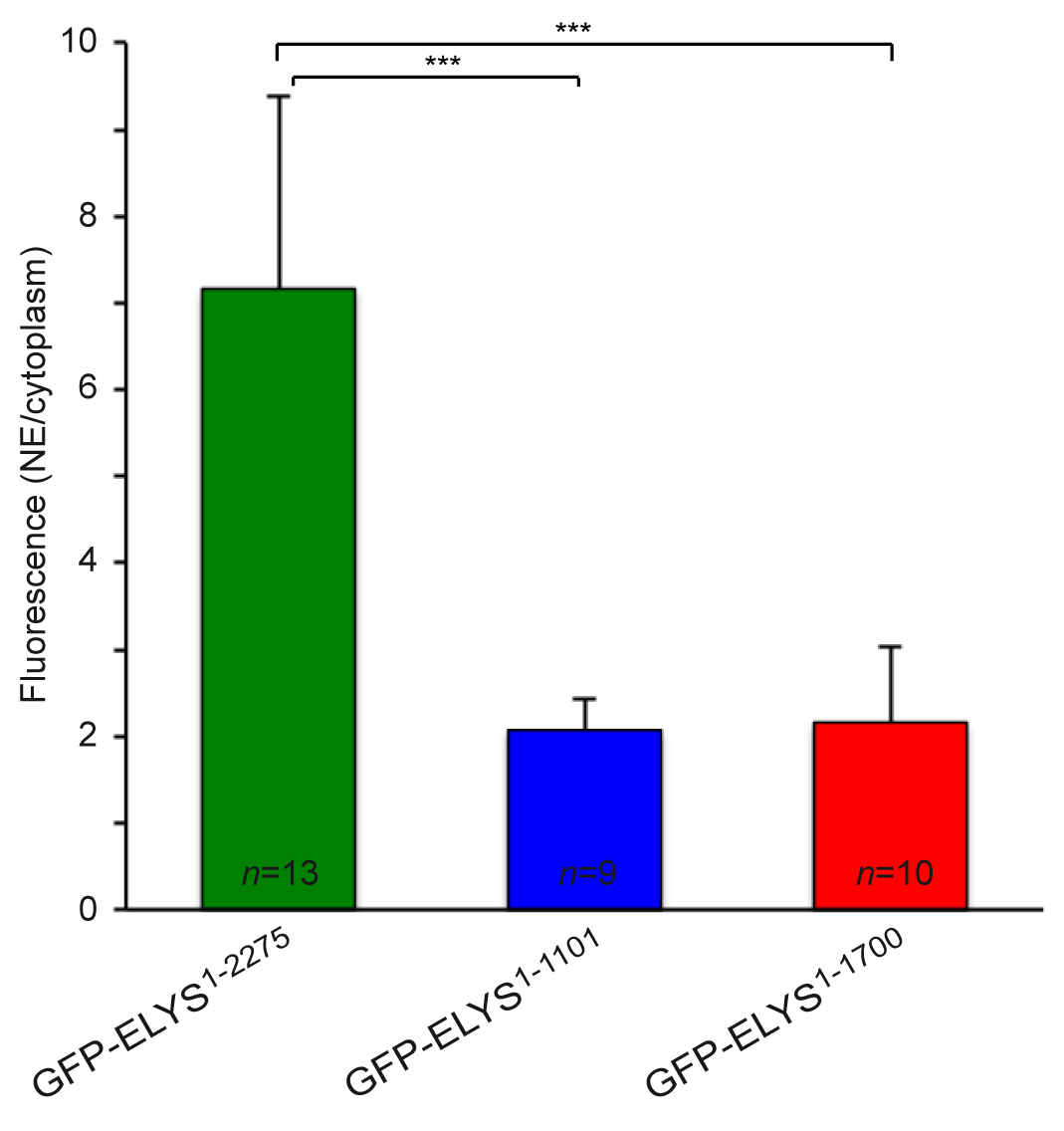

Supplement: S6 Fig — Fluorescence intensity of the NE and cytoplasm was determined for HeLa cells transiently expressing GFP fused to full-length ELYS (ELYS1-2275), ELYS1-1101, or ELYS1-1700. The ratio of NE versus cytoplasmic fluorescence was reduced by 70–71% for the two truncated ELYS proteins. *** p<0.001 by unpaired two-tailed t-test. (TIF) [file pgen.1006131.s006.tif]
